# Induction of hormesis in plants by urban trace metal pollution

**DOI:** 10.1038/s41598-021-99657-3

**Published:** 2021-10-13

**Authors:** Mirko Salinitro, Gaia Mattarello, Giorgia Guardigli, Mihaela Odajiu, Annalisa Tassoni

**Affiliations:** Department of Biological, Geological and Environmental Sciences, Alma Mater Studiorum-University of Bologna, Via Irnerio 42, 40126 Bologna, Italy

**Keywords:** Plant stress responses, Abiotic, Plant sciences, Environmental sciences

## Abstract

Hormesis is a dose–response phenomenon observed in numerous living organisms, caused by low levels of a large number of stressors, among which metal ions. In cities, metal levels are usually below toxicity limits for most plant species, however, it is of primary importance to understand whether urban metal pollution can threaten plant survival, or, conversely, be beneficial by triggering hormesis. The effects of Cd, Cr and Pb urban concentrations were tested in hydroponics on three annual plants, *Cardamine hirsuta* L.*, Poa annua* L. and *Stellaria media* (L.) Vill., commonly growing in cities. Results highlighted for the first time that average urban trace metal concentrations do not hinder plant growth but cause instead hormesis, leading to a considerable increase in plant performance (e.g., two to five-fold higher shoot biomass with Cd and Cr). The present findings, show that city habitats are more suitable for plants than previously assumed, and that what is generally considered to be detrimental to plants, such as trace metals, could instead be exactly the plus factor allowing urban plants to thrive.

## Introduction

The term hormesis describes the biphasic response to a large spectrum of compounds observed in numerous living organisms (bacteria, plants, animals, etc.) and characterized by opposite effects exerted by low and high doses of the same substance^1–3^. It is assumed that hormesis is an adaptive response to stress, possibly triggered by an initial disruption of homeostasis by low levels of biotic or abiotic stressors, often followed by a process of overcompensation aimed at re-establishing the previous status^1^ and at protecting the organism through the stimulation of cellular defence mechanisms^4,5^. However, behind this mechanistic interpretation there are complex reactions that still need validation^6^. The induction of this adaptive response in plants, involves several steps such as perception and transduction of the stress-signal, and stimulation of the hormetic response at the transcriptional and post-transcriptional level^7,8^.

When an organism exhibits a hormetic response, this can be described either by a U-shaped (or J-shaped) or by an inverted U-shaped (or inverted J-shaped) curve, depending on the measured endpoints. If the endpoints are dysfunctional (such as for carcinogenesis and disease incidence), there will be a higher incidence in the control and at high levels of the tested substance, whereas it will be minimal at low doses, thus resulting in a U-shaped curve. Conversely, if the endpoints are related to normal functions (such as fertility or growth), an increase at low doses of the tested substance and a decrease in the control and at high doses will be detected, resulting in an inverted U-shaped curve^8,9^.

Evidence of hormesis has been widely reported in the medical field^10–12^ and plant sciences^13^. Different physical or chemical agents have been shown to cause hormesis in plants, such as organic compounds (e.g., weedkillers, formaldehyde), biological molecules (e.g., polyphenols), physical stressors (e.g., temperature, radiations) and metals^5,14–19^. Furthermore, it was hypothesised a practical application of hormesis in agriculture, e.g., by making plants more resistant to adverse conditions (such as soil pollution and drought) while improving their productivity^5^. For instance herbicides applied at very low concentrations, may increase plant growth^20^ and seed yield^21^, regulate the production of auxins, improve cation transport in the rhizosphere^22^ and stimulate CO_2_ assimilation, transpiration, stomatal conductance and electron transport^23^. However hormesis-based interventions, by using sub-lethal doses of weedkillers as bio-stimulants applied at field scale^5^, must take into consideration possible side effects, like overdosing and the quality and safety of the final product^24^. Hormetic effects have also been reported for a large number of plant taxa^19,25,26^ as a reaction to low concentrations of metals, such as lanthanum, cadmium, chromium, which have been widely studied for their acute toxicity^8,27^. For instance, 56 μM lanthanum (La) positively affected numerous biological parameters in plants, like biomass production, cell growth rate, chlorophyll content, peroxidase activity and flavonoid content^9,19,27^. Similarly, the non-essential nutrient cadmium (Cd) has been demonstrated to increase the dry biomass of *Lonicera japonica* Thunb. by around 20–40%^9^.

Some main action mechanisms have been identified to be at the base of plant hormetic responses triggered by metal ions^28,29^: (i) ionic interactions between different chemicals present in the soil (or in the liquid nutrient solution) that can affect nutrient absorption in a positive or negative way; (ii) metal-induced specific defence reactions, such as activation of metal tolerance genes; (iii) metal-induced general defence reactions, triggered by the generation of reactive oxygen species (ROS) and leading to the activation of the antioxidant response, (iv) general increase in photosynthetic system efficiency, which determines the final hormetic stimulating effect (e.g., biomass increase).

Moderate levels of metal pollution are widespread in our cities, exposing plants growing in urban environments often to this type of stress. However, metal levels in urban soils are usually below the toxicity limits for most plant species (i.e., 0.4–0.8 mg/kg DW for Cd, 20–100 mg/kg DW for Pb, and 30–200 mg/kg DW for Cr)^30–32^. In addition, urban soils are often characterized by high pH and organic matter content, causing a strong reduction of metal ions availability.

Because of their low concentration and availability, it can be hypothesized that these metals not only do not cause harm to plants, but conversely can trigger hormetic responses enhancing plant performances. Consequently, the aim of the present study was to assess whether Cd, chromium (Cr), and lead (Pb) at average urban concentrations can cause hormetic responses in plants. To test this hypothesis, plants of three weeds common to all urban habitats, *Cardamine hirsuta* L.*, Poa annua* L. and *Stellaria media* (L.) Vill*,* were grown hydroponically with low doses of the chosen metal pollutants and their traits evaluated for hormetic responses. The hydroponic approach was chosen to exclude metal-soil interactions, that could cause uncontrolled changes in metal availability due to pH shifts, organic matter content and adsorption by soil particles. This way, it was possible to better link the hormetic response to the actual metal concentration in the nutrient solution.

## Materials and methods

### Species selection

**Figure 1:**
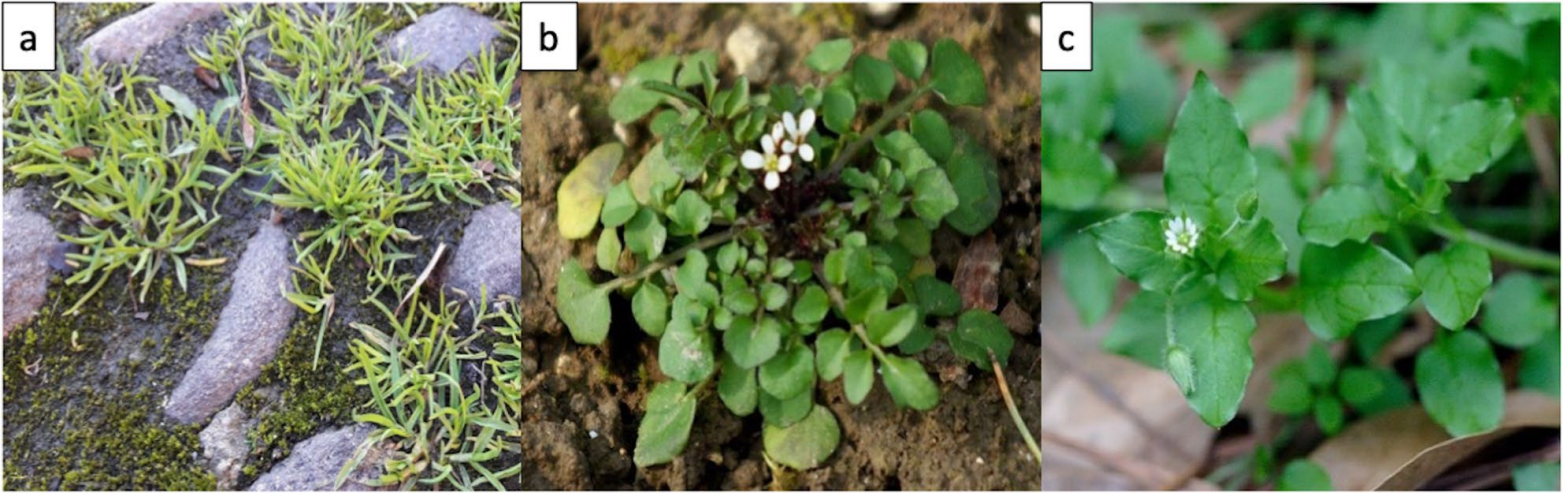
The three species used in the present study: (**a**) *Poa annua* L., (**b**) *Cardamine hirsuta* L., (**c**) *Stellaria media* (L.) Vill. Photographs by M. Salinitro.

For this study, three common annual species, *Poa annua* L. (Poaceae family, Fig. 1a)*, Cardamine hirsuta* L. (Brassicaceae family, Fig. 1b) and *Stellaria media* (L.) Vill. (Caryophyllaceae family, Fig. 1c), were selected for their fast-growing habit, easiness of recognition and for widespread presence in urban habitats. Seeds of these species were collected in the Ticino Natural Park (Loc. Besate, Milan, Italy). This area is characterized by undisturbed sandy soil, with low organic matter content, minimal anthropic pollution and low metal concentrations^33^. The collection location was selected to avoid biases caused by plant populations that might have evolved metal tolerance or metal pre-adaptation. No permits were necessary for the collection of seeds and specimens of the selected species, since these plants are cosmopolitan species of no conservation interest and the collection site was outside of any protected area. The formal identification of the used species was carried out by M. Salinitro in collaboration with experts of the Botanical Garden of Bologna’s University. Voucher specimens of the selected species were collected and deposited at the Herbarium of the University of Bologna. The present study complies with relevant institutional, national, and international guidelines and legislation.

### Plant growth and sample collection

Seeds were sown on a medium composed of 50% compost and 50% coarse sand and then placed for 1 week at 4 °C for cold stratification. After stratification, seeds were kept at 22 °C with a 16–8 h light–dark photoperiod until germination. One week after germination, seedlings were transferred to the hydroponic system composed of six 18 L-plastic tanks, one used as a control and five dedicated to metal treatments^34^. 15 plants (5 replicates per species) were grown together in each tank, thus subjected to interspecific and intraspecific competition. Half-strength Hoagland’s solution^35^ was used as hydroponic nutrient solution (pH 6.0 ± 0.1). A 10% v/v replacement of the nutrient solution was performed every 2 days and the pH re-adjusted. Tanks were kept at 22 ± 1 °C and a 16–8 h light–dark photoperiod.

Five concentrations of Cd, Cr and Pb were selected based on the average urban levels reported for two Italian cities^30^ and tested to assess their capacity to stimulate hormesis in the selected species. Cadmium was tested at 0.5 µM, 0. 75 µM, 1 µM, 1.5 µM, and 2 µM concentrations; chromium at 5 μM, 10 μM, 25 μM, 50 μM, and 100 μM; and lead at 0.5 µM, 1 µM, 5 µM, 7.5 µM, and 15 µM. To spike each tank with the proper amount of metal, 0.1 M CdCl_2_ * 2.5H_2_O, 0.1 M CrCl_3_ * 6H_2_O, 0.1 M Pb-EDTA stock solutions were used.

Plants were cultivated hydroponically for 4 weeks, a time period sufficient to allow the maximum vegetative development of plants before the beginning of flowering. During harvesting, shoots and roots were collected separately, grinded in liquid nitrogen to obtain a homogeneous powder and stored at − 80 °C. Sample aliquots (0.5 g of fresh weight, gFW) were oven-dried at 60 °C for 48 h until constant weight, and the amount of dry weight was determined (g of dry weight, g DW).

### Trace metal quantification

Root or shoot powder (0.3 g DW) were placed in TFM (modified Polytetrafluoroethylene) digestion tubes for close vessel digestion, together with 6 ml of 69% (v/v) HNO_3_ and 0.5 ml of 35% (v/v) H_2_O_2_ (modified method from Tüzen^36^). The samples were subsequently subjected to a microwave digestion cycle of: 2 min at 250 W, 2 min at 400 W, 1 min at 0 W and 2 min at 600 W, and 33 min cooling. The quantification of total elements was carried out with a Spectro Arcos ICP-OES (inductively coupled plasma optical emission spectroscopy) (Ametek, Berwyn, Pennsylvania, US). The limits of quantification of the analyzed elements were 0.000373 mg/kg DW for Cd, 0.00042 mg/kg DW for Cr, 0.0017 mg/kg DW for Pb.

### Plant traits measurement

**Figure 2:**
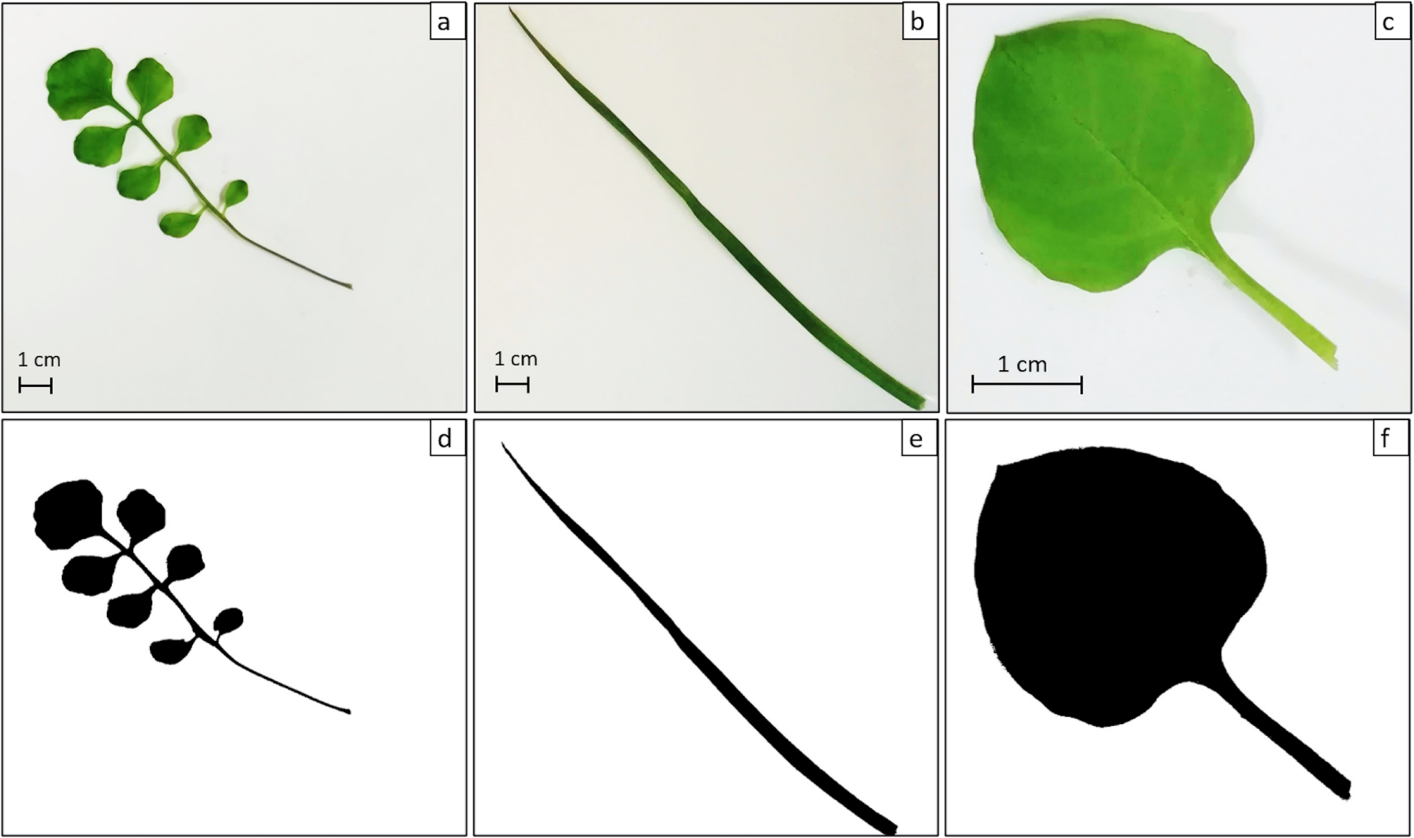
Binary transformation of leaves used to calculate leaf area, in the three tested species: (**a**–**d**) *Cardamine hirsuta*; (**b**,**e**) *Poa annua*; (**c**–**f**) *Stellaria media*. Photographs by M. Salinitro.

After 4 weeks of cultivation, the number of nodes and the leaf area of each plant were measured. Plant node number was calculated differently depending on the species: in *P. annua* it was equated to the number of tillers generated by the primary plant; in *C. hirsuta* and *S. media* every node corresponded to the insertion point of 1 or 2 leaves, respectively. Nodes of lateral branches were never counted. To measure the leaf area, the first fully developed leaf (starting from the apex) of the main sprout was measured for all species. The chosen leaves were removed from the plant with their stalks and placed on a background cardboard square of known size (5 × 5 cm or 15 × 15 cm) under a transparent plastic film to prevent wrinkles. Pictures were taken with a camera and the leaf area was calculated with ImageJ software (https://imagej.nih.gov/ij/download.html). Every picture, cut at the dimension of the cardboard square, was converted to binary (only black and white pixels) to obtain a black leaf shape on a white background of known area (Fig. 2), white and black pixels were counted, and the leaf area was calculated as follows:

$${\text{Leaf}}\;{\text{area}}\;\left( {{\text{cm}}^{2} } \right) = {{{\text{black}}\;{\text{pixels}}*{\text{cardboard}}\;{\text{area}}\left( {{\text{cm}}^{2} } \right)} \mathord{\left/ {\vphantom {{{\text{black}}\;{\text{pixels}}*{\text{cardboard}}\;{\text{area}}\left( {{\text{cm}}^{2} } \right)} {{\text{total}}\;{\text{pixels}}}}} \right. \kern-\nulldelimiterspace} {{\text{total}}\;{\text{pixels}}}}$$

### Photosynthetic pigment quantification

Total amount of photosynthetic pigments (sum of chlorophylls a and b and total carotenoids) was determined starting from 0.1 gFW of grinded plant shoots^34^.

### Data analysis

Statistical analyses were performed using R software version 4.0.2. (https://cran.r-project.org/bin/windows/base/). Data were organized in 3 datasets composed of 13 variables (columns) and 90 observations (rows), as in Supplementary Tables 1–3. The differences in metal uptake and plant growth parameters were evaluated for each tested metal, among the different treatments. The statistical analysis was performed using 5 plants replicates all grown in the same treatment tank. Data were tested for normality using the Shapiro–Wilk normality test, and for homogeneity using the Levene’s test for homogeneity of variance, with default parameters from the package *car* (https://CRAN.R-project.org/package=car).

For parametric data, one-way ANOVA (p ≤ 0.05) followed by the post hoc Tukey HSD test (two-tailed) was performed to detect significant differences among groups. ANOVA p-values*,* f-values*,* and degrees of freedom are provided in the Supplementary Table 4, while p-values from post-hoc tests are reported in the results section where needed. A polynomial regression was applied to root and shoot dry weigh/plant and number of nodes/plant variables, to graphically visualize the hormetic curve on plotted data. All graphical elaborations were performed using the R package *ggpubr* (https://CRAN.R-project.org/package=ggpubr).

## Results

### Metal uptake

The uptake of Cd, Cr, and Pb in the three studied species after 4 weeks of hydroponic culture was determined in both roots and shoots (Table 1). Overall, in both organs, the amount of these metals increased with their increasing concentration in the nutrient solution (Table 1). The highest Cd accumulation in roots was observed at 2 μM in *S. media* (67.88 mg/kg DW), while in shoots the highest Cd concentration was found in *P. annua* (13.75 mg/kg DW). *C. hirsuta* and *S. media* showed lower amounts of Cd in shoots with maximum levels of 5.45 and 4.51 mg/kg DW, respectively. Cr accumulated most in *C. hirsuta* roots (20.25 mg /kg DW) and in *S. media* aerial parts (38.37 mg/kg DW) of plants subjected to 100 μM Cr. Finally, the highest Pb content was detected in *P. annua* roots (15.64 mg /kg DW) treated with 15 μM Pb and in *C. hirsuta* shoots treated with 7.5–15 μM Pb (average of 5.80 mg/kg DW).

**Table 1:** Metal accumulation (mg / kg DW) in roots and shoots of the three tested species.

Species	Treatment Cd (μM)	Root (mg/kg DW)	Shoot (mg/kg DW)	Treatment Cr (μM)	Root (mg/kg DW)	Shoot (mg/kg DW)	Treatment Pb (μM)	Root (mg/kg DW)	Shoot (mg/kg DW)
*Cardamine hirsuta*	Control (0)	< LoD^a^	< LoD^a^	Control (0)	0.075 ± 0.003^a^	3.78 ± 0.50^a^	Control (0)	< LoD^a^	< LoD^a^
	0.5	< LoD^a^	< LoD^a^	5	0.737 ± 0.091^a^	3.81 ± 0.31^a^	0.5	< LoD^a^	< LoD^a^
	0.75	0.025 ± 0.004^a^	1.31 ± 0.26^b^	10	1.76 ± 0.15^b^	5.10 ± 1.90^a^	1	0.058 ± 0.013^a^	1.57 ± 0.32^b^
	1	0.027 ± 0.007^a^	3.44 ± 0.36^c^	25	3.32 ± 0.16^c^	6.15 ± 0.52^a^	5	0.190 ± 0.027^b^	3.93 ± 0.49^c^
	1.5	0.045 ± 0.003^b^	5.18 ± 0.35^d^	50	7.65 ± 0.36^d^	8.47 ± 0.59^b^	7.5	0.233 ± 0.032^b^	6.09 ± 0.27^d^
	2	0.044 ± 0.006^b^	5.45 ± 0.39^d^	100	20.25 ± 0.77^e^	15.18 ± 2.33^c^	15	0.503 ± 0.089^c^	5.48 ± 0.40^e^
*Poa annua*	Control (0)	< LoD^a^	< LoD^a^	Control (0)	0.053 ± 0.014^a^	1.69 ± 0.26^a^	Control (0)	< LoD^a^	< LoD^a^
	0.5	1.97 ± 0.48^b^	0.99 ± 0.19^b^	5	0.46 ± 0.05^b^	1.68 ± 0.26^a^	0.5	0.993 ± 0.658^b^	< LoD^a^
	0.75	3.16 ± 0.15^b^	1.46 ± 0.19^b^	10	1.09 ± 0.11^c^	2.63 ± 0.20^a^	1	2.50 ± 0.54^c^	< LoD^a^
	1	5.90 ± 0.37^c^	4.51 ± 0.53^c^	25	2.43 ± 0.12^d^	8.85 ± 0.95^b^	5	4.52 ± 0.49^d^	0.206 ± 0.020^b^
	1.5	9.92 ± 0.18^d^	7.52 ± 0.57^d^	50	2.73 ± 0.15^d^	12.52 ± 0.99^c^	7.5	7.57 ± 1.40^e^	0.541 ± 0.098^c^
	2	19.18 ± 1.68^e^	13.75 ± 0.96^e^	100	10.36 ± 0.43^e^	13.05 ± 0.86^c^	15	15.64 ± 2.26f.	1.27 ± 0.18^d^
*Stellaria media*	Control (0)	< LoD^a^	< LoD^a^	Control (0)	0.008 ± 0.001^a^	< LoD^a^	Control (0)	< LoD^a^	< LoD^a^
	0.5	0.068 ± 0.010^a^	< LoD^a^	5	0.54 ± 0.03^a^	< LoD^a^	0.5	< LoD^a^	0.068 ± 0.010^a^
	0.75	0.07 ± 0.01^a^	1.75 ± 0.04^b^	10	1.21 ± 0.09^b^	2.95 ± 0.75^b^	1	0.167 ± 0.058^a^	0.677 ± 0.329^a^
	1	34.09 ± 6.41^b^	2.77 ± 0.29^c^	25	2.62 ± 0.25^c^	9.02 ± 0.89^c^	5	3.22 ± 0.33^b^	2.47 ± 0.49^b^
	1.5	51.00 ± 2.00^c^	2.97 ± 0.51^c^	50	3.84 ± 0.07^d^	25.54 ± 1.59^d^	7.5	5.11 ± 0.26^c^	4.18 ± 0.34^c^
	2	67.88 ± 4.21^d^	4.51 ± 0.28^d^	100	16.37 ± 0.64^e^	38.37 ± 3.73^e^	15	6.80 ± 0.42^d^	4.89 ± 0.43^d^

Each value is the average of 5 biological replicates (n = 5) ± S.D (see detailed data met_root and met_shoot in Supplementary Tables 1–3). LoD, Limit of detection. Different letters indicate differences among groups after one-way ANOVA (*p* ≤ 0.05) (Supplementary Table 4) followed by post-hoc Tukey HSD test.

All species showed a bioaccumulation factor (BAF, shoot metal concentration/nutrient solution metal concentration) higher than 1, indicating an active uptake of these metals from the nutrient solution, followed by an accumulation in plant organs. Cd-treated plants showed the highest BAF values (on average 25.3, 36.1 and 20.7, respectively, for *C. hirsuta, P. annua* and *S. media*). Instead, BAF values were on average much lower for *C. hirsuta, P. annua* and *S. media* plants treated with Cr (7.1, 5.1, and 5.4, respectively) and Pb (4.2, 0.3 and 2.1, respectively).

Metal translocation from roots to shoots was particularly abundant in *C. hirsuta*, which showed average translocation factor (TF, shoot metal concentration/root metal concentration) values of 109.3 and 21.6, respectively, for Cd and Pb, while *P. annua* and *S. media* were less efficient with average TF values of 0.64 and 0.07, respectively, for Cd, and of 6.5 and 1.6, respectively, for Pb. Cr translocated at a similar rate in all three species with an overall average TF value of 3.0.

### Hormesis induced by cadmium

**Figure 3:**
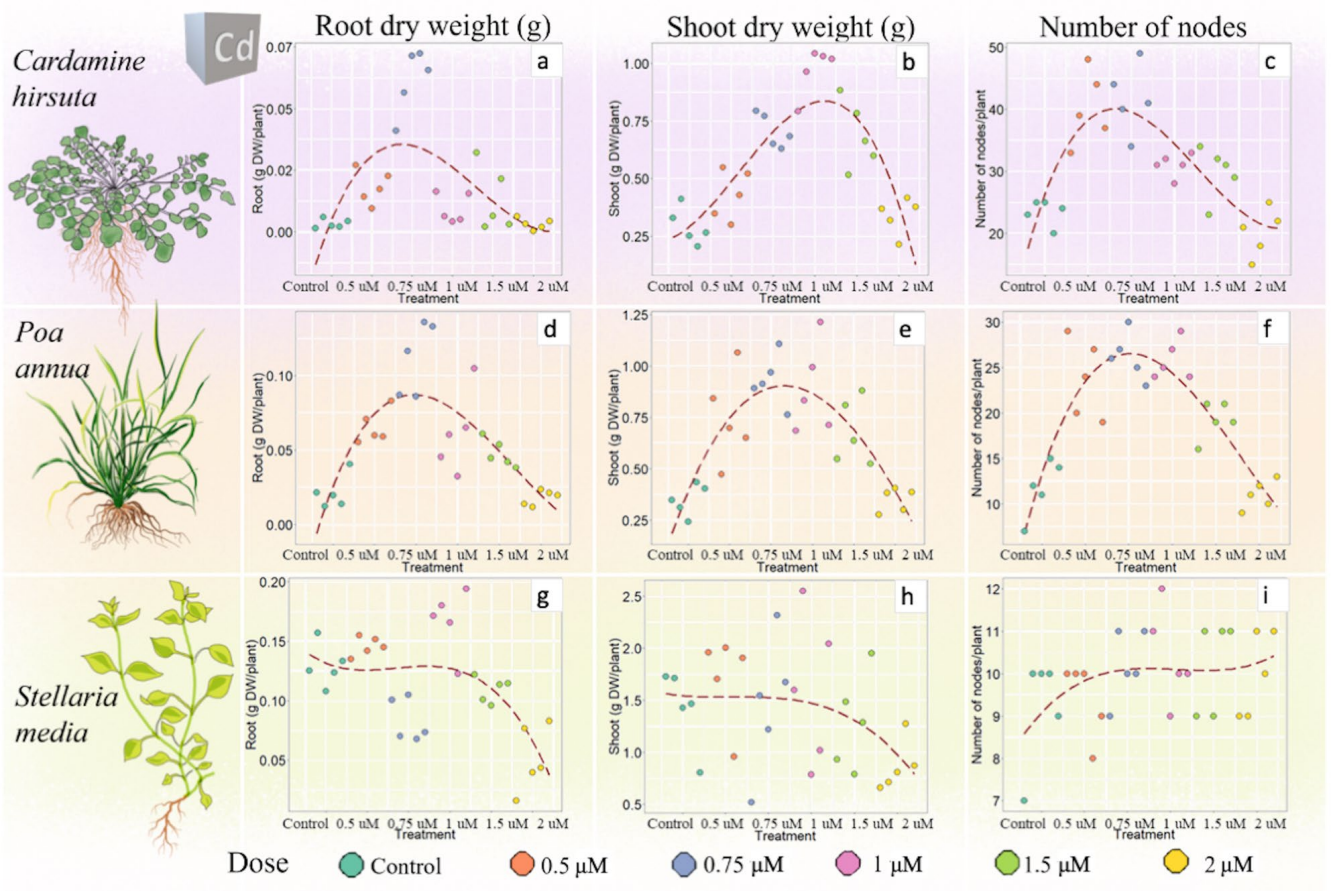
Effect of cadmium on root and shoot dry biomass (g DW/plant) and number of nodes per plant in *Cardamine hirsuta* (**a**–**c**) *Poa annua* (**d**–**f**) and *Stellaria media* (**g**–**i**)*.* A polynomial regression (dashed line) was applied to visualize the presence/absence of a hormetic curve. Each treatment is represented by 5 biological replicates (n = 5) (Supplementary Table 1). Drawings of plants by G. Mattarello.

The tested Cd concentrations induced a marked hormetic response in *C. hirsuta* and *P. annua*, showing a strong influence on root and shoot dry biomass per plant and on the number of nodes (Fig. 3). In particular, *C. hirsuta* root dry weight/plant (Fig. 3a) increased about 20-fold from control (0.003 g DW/plant) to 0.75 μM Cd (0.06 g DW/plant) (*p* ≤ 0.01), to return to control levels (0.003 g DW/plant) at 2 μM Cd. A similar pattern was observed for *C. hirsuta* shoot dry weight/plant (Fig. 3b), which showed an average three-fold higher value at 1 μM Cd (0.97 g DW/plant), compared to the control (0.29 g DW/plant) and 2 μM Cd (0.34 g DW/plant) (*p* ≤ 0.01). In both species a significant trend in root/shoot DW ratio was observed. In *C. hirsuta* root/shoot ratio was 0.9 at 0.75 μM Cd compared to the 0.01 in control and 2 μM Cd samples, similarly in *P. annua* the ratio was 0.1 in 0.75 1 μM Cd compared to 0.06 in control and 2 μM Cd samples. The number of nodes per plant almost doubled at 0.75 μM Cd (41.6 nodes/plant) compared to the control (23.4 nodes/plant) and 2 μM Cd (20.2 nodes/plant) (*p* ≤ 0.01) (Fig. 3c). Cd treatments did not affect total photosynthetic pigments content and leaf area (averages of 160 mg/kg FW and 14.7 cm^2^, respectively, details in Supplementary Table 1).

*Poa annua* was largely affected by Cd treatments, expressing a marked increase of all measured morphological traits (root and shoot dry weight/plant, number of nodes, leaf area, total amount of photosynthetic pigments) at Cd concentrations between 0.5 and 1 μM, showing a typical reverse U-shaped curve (Fig. 3d–f, Supplementary Table 1). On the other hand, *S. media* did not show a specific hormetic response linked to Cd treatments for any of the analysed variables (Fig. 3g–i, Supplementary Table 1).

### Hormesis induced by chromium

**Figure 4:**
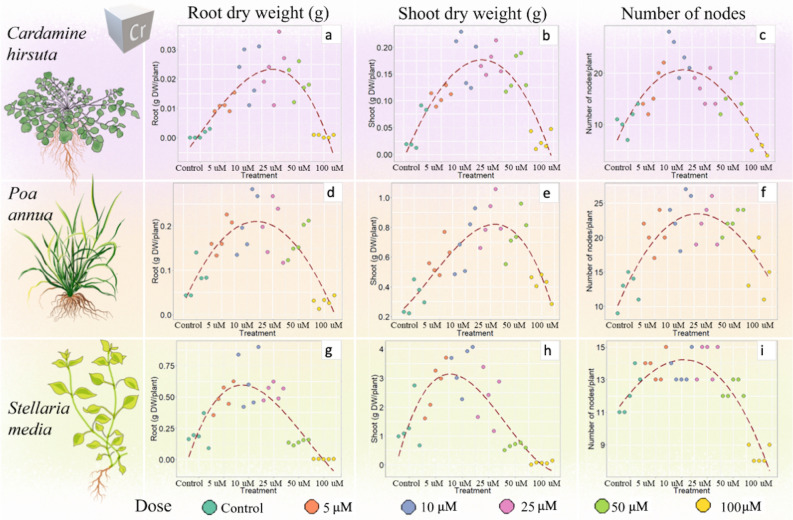
Effect of chromium on root and shoot dry biomass (g DW/plant) and number of nodes per plant in *Cardamine hirsuta* (**a**–**c**), *Poa annua* (**d**–**f**) and *Stellaria media* (**g**–**i**). A polynomial regression (dashed line) was applied to visualize the presence/absence of a hormetic curve. Each treatment is represented by 5 biological replicates (n = 5) (Supplementary Table 2). Drawings of plants by G. Mattarello.

Hormesis induced by chromium was observed in all tested species, with a two to four-fold increase with respect to the control in shoot and root dry weight/plant at concentrations between 10 to 50 μM (Fig. 4).

Root dry weight in *C. hirsuta* (Fig. 4a), for example, ranged from 0.003 g DW/plant in the control to an average of 0.023 g DW/plant at Cr concentrations between 10 and 50 μM, to decrease again to 0.001 g DW/plant at 100 μM Cr (*p* ≤ 0.01). Shoot biomass and number of nodes per plant showed similar patterns (Fig. 4b,c, Supplementary Table 2). Conversely, total photosynthetic pigments and leaf area were not significantly influenced by Cr with averages of 146.3 mg/kg FW and 0.6 cm^2^, respectively, in all treatments.

Cr treatment also affected *P. annua* growth, with a marked hormetic effect on root and shoot dry weight/plant and number of nodes observed at intermediate Cr concentrations (10–50 μM) (Fig. 4d–f, details in Supplementary Table 2). The highest content of photosynthetic pigments was detected in control and 100 μM Cr samples (181.3 mg/kg FW), while lower values were measured at 10–25 μM concentrations (average of 142.1 mg/kg FW) (*p* ≤ 0.01). Leaf area varied among treatments with no clear trends.

*S. media* showed a marked hormetic effect but also toxicity symptoms in response to Cr. In fact, plants subjected to 100 μM Cr exhibited toxicity, with lower values of root and shoot biomass and number of nodes than the control (Fig. 4g–i, Supplementary Table 2). On the contrary, total photosynthetic pigments content increased in plants grown at Cr concentrations of 50 and 100 μM to levels (average 108.2 mg/kg FW) comparable to the control (128.9 mg/kg FW) (*p* ≤ 0.05). Leaf area was not affected by Cr treatments (Supplementary Table 2).

### Hormesis induced by lead

**Figure 5:**
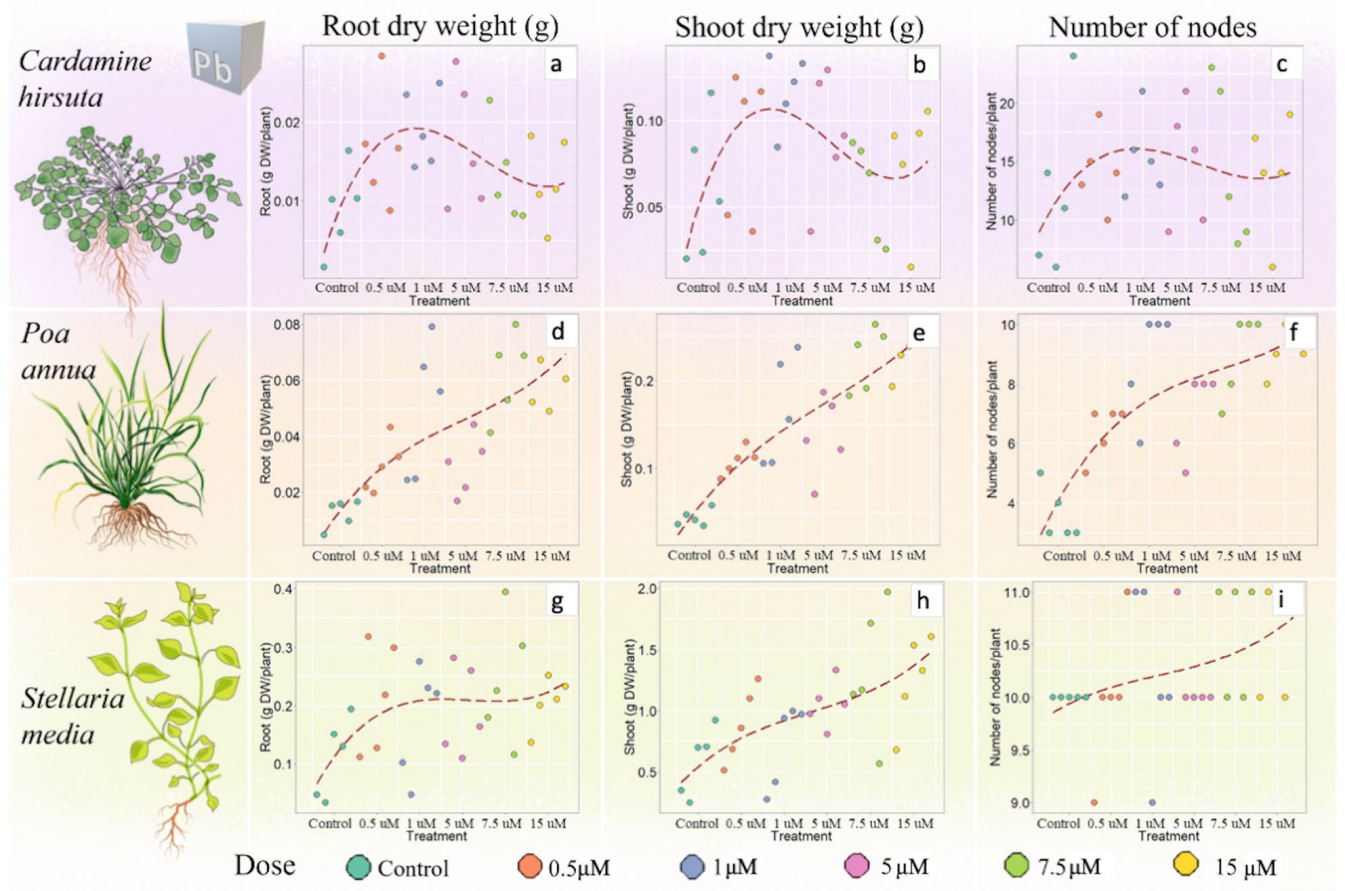
Effect of lead on root and shoot dry biomass (g DW/plant) and number of nodes per plant in *Cardamine hirsuta* (**a**–**c**), *Poa annua* (**d**–**f**) and *Stellaria media* (**g**–**i**). A polynomial regression (dashed line) was applied to visualize the presence/absence of a hormetic curve. Each treatment is represented by 5 biological replicates (n = 5) (Supplementary Table 3). Drawings of plants by G. Mattarello.

At the tested concentrations, Pb did not induce hormesis in any of the studied species, however, plant growth was affected in different ways. In *C. hirsuta*, concentrations between 1 and 5 μM slightly increased root and shoot dry weight (+ 32% and + 21%, respectively) and node number (+ 15%) (Fig. 5a–c, Supplementary Table 3) compared to control conditions. However, when tested with ANOVA, these differences resulted non-significant (*p* = 0.162, *p* = 0.119, and *p* = 0.968, respectively).

In *P. annua****,*** the stimulatory effect on the three analysed variables ranged from two to fivefold increases in plants treated with Pb concentrations between 7.5 and 15 μM (*p* ≤ 0.01) (Fig. 5d–f). A similar pattern was detected in *S. media*, although the stimulatory effect was lower compared to that of *P. annua* and limited to shoot dry weight/plant in 15 μM Pb-treated plants (+ 115% compared to the control, Fig. 5h). Conversely, in *S. media* the number of nodes (Fig. 5i) was not affected by Pb treatment, with an average of 10.2 nodes/plant in all treatments. Total photosynthetic pigments content and leaf area did not vary under the different Pb treatments, with average values of 107.57 mg/kg FW and of 5.85 cm^2^, respectively (Supplementary Table 3).

## Discussion

**Figure 6:**
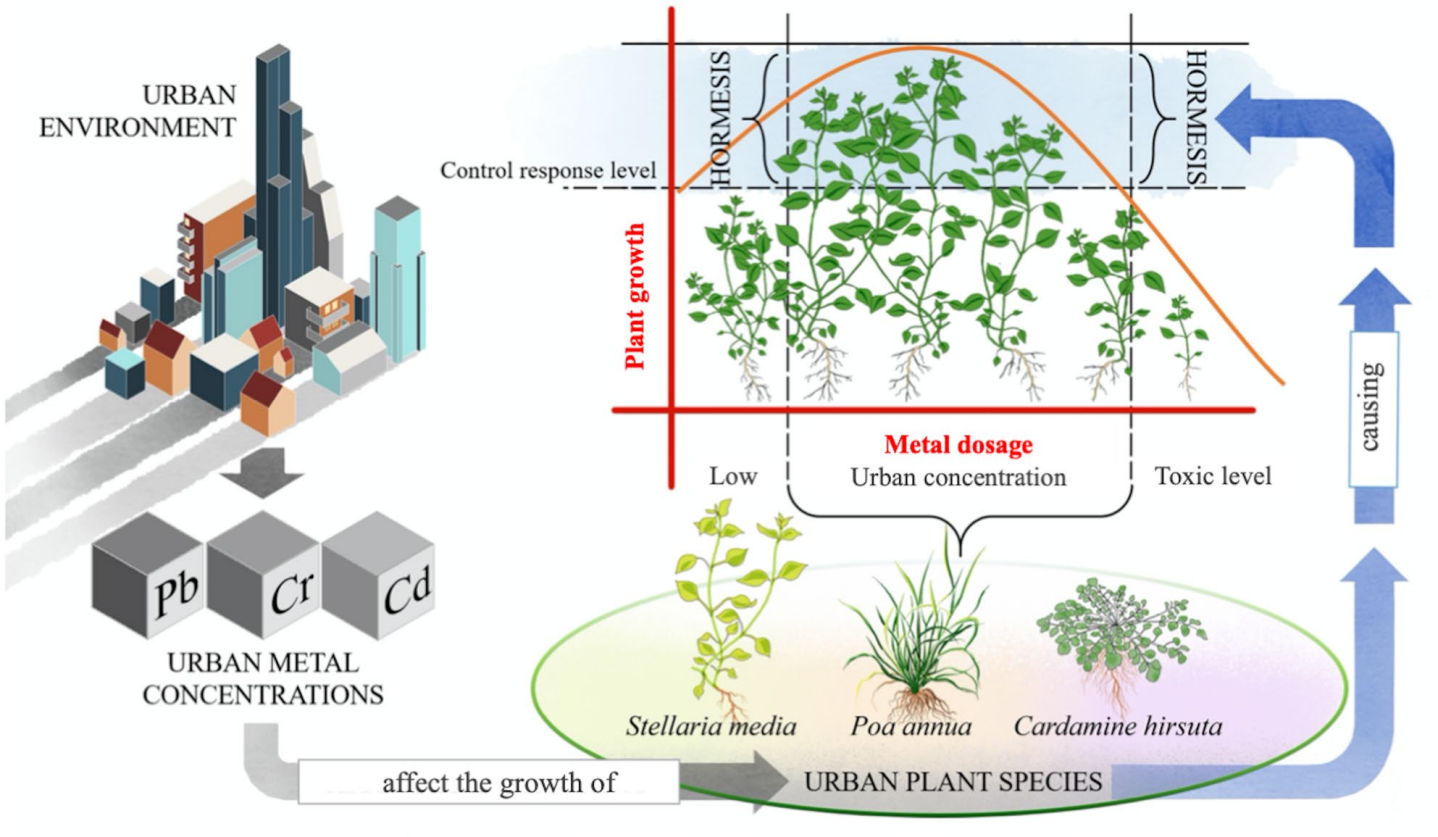
Proposed hormesis effect caused by urban metals on plant growth. Drawings by G. Mattarello.

The presented data demonstrated that low concentrations of the toxic metals Cd, Cr and Pb, comparable to those found in urban soils, can trigger hormesis at various degrees in *P. annua*, *S. media* and *C. hirsuta.* The presence of low levels of toxic metals in urban soils could, therefore, make these plant species more resilient and able to survive in anthropic environments (this concept is summarised in Fig. 6). To better highlight only the effects caused by the supplied stressors, plants were grown in hydroponic culture allowing for a strict control of metal availability and nutrient supply in the liquid medium and avoiding experimental biases due to element sequestration or pH changes that could happen when working with soil^37^.

Overall, Cr induced the most striking hormetic response (hormesis observed in all species) (Fig. 4), followed by Cd (hormesis observed in *C. hirsuta* and *S. media*) (Fig. 3), while Pb did not cause a clear hormetic response. In fact, despite a biomass stimulation observed in all species in the presence of Pb, only *P. annua* and *S. media*, showed what was interpreted as the first half of a conceivable inverted U-shaped hormetic curve (Fig. 5).

The effects of the three tested metals varied substantially among species, causing hormesis at different levels. This trend has previously been observed, suggesting that the same hormetic dose of different stressors (e.g., glyphosate or Cd micro-doses) can induce both stimulatory and inhibitory effects depending on the plant species^8,38^.

Biomass increase was the clearest sign of hormesis occurring, in fact, when treated with 0.75 μM Cd, *C. hirsuta* shoot biomass more than doubled, while root biomass increased almost 20 times compared to control treatment. A similar effect was observed in *P. annua*, with shoot and root biomass increasing 1.5 and 5 times, respectively, when kept at 0.75 μM Cd compared to control and 2 μM treatments (Fig. 3d,e). In agreement with our findings, a substantial biomass increase was reported for *Spirodela polyrrhiza* L. treated with 1 μM Cd^25^.

In general, cadmium preferentially accumulated in roots (i.e., averages of 3.7 mg/kg DW in roots and 2.3 mg/kg DW in shoots of *P. annua* treated with 0.5-1 µM Cd, Table 1), which could partially explain the general stronger biomass increase in roots compared to shoots (Fig. 3d). A general root volume, biomass and length increase was reported for several species under Cd treatment^29^. Similarly, studies on *Camellia sinensis* demonstrated that the hormetic effect was more pronounced in roots compared to shoots (74% and 27% biomass increase, respectively) and proportional to the metal concentration detected in these organs^26^.

The 100% increase in the number of nodes per plant produced after 4 weeks of cultivation of *C. hirsuta* and *P. annua* demonstrated a higher growth rate in plants treated with 0.5–1 µM Cd compared to control and 2 µM Cd treatments (Fig. 3f). Similarly, it was observed that root elongation in *Brassica napus* treated with 5 to 15 μM Cd was faster compared to untreated plants^39^. The presented data, therefore, confirm that plants treated with hormetic doses of a stressor increase cell division and cell elongation rate, possibly due to reduced cell wall rigidity as previously reported^22,40^.

In *P. annua,* despite the better performance in biomass production, Cd had negative effects on the production of photosynthetic pigments, which was higher in control treatment but steadily decreased with increasing Cd concentrations. In contrast with our findings, the biomass increase is usually associated with a higher chlorophyll content and enhanced efficiency of the photosynthetic system^29^. However, similar results were reported for *Spirodela polyrrhiza* plants, which showed a reduction in photosynthetic pigment content when treated with Cd concentrations above 0.5 µM, although biomass production increased^25^. It can be speculated that Cd-induced hormesis was the consequence of an over-compensatory response, which did not completely recover the damage caused by the metal. In fact, it has been widely reported^41,42^ that plant exposure to Cd strongly affects chlorophyll and carotenoid concentrations in leaves, as this metal can competitively bind to the Ca-binding sites of PSII, inactivating the water-splitting system. According to Calabrese^2^, the reaction to damage occurring in one part of the plant can influence the growth of other sections of the organism. Therefore, even if the plant fails in repairing the damage (i.e., chlorophyll suppression), the action undertaken could have benefits for other plant functions, as was here observed for *P. annua* under Cd exposure (Fig. 3). Generation of ROS linked to metal-related oxidative stress could be identified as responsible for the hormetic over-compensatory response^25,26,28^. In fact, increased plant growth was reported to be connected to ROS production, in particular hydrogen peroxide, which acts as a signalling molecule allowing inward water transport in young cells and causing cell expansion^5^.

Exposure to low doses of Cr clearly had beneficial effects in all the tested species, nonetheless, this metal caused toxicity at concentrations around 100 μM (Fig. 4). A complete inverse U-shaped hormetic curve was observed, going from no effects in the control to beneficial effects at intermediate concentrations (5–50 μM Cr), followed by toxicity at 100 μM Cr. In particular, toxicity given by the highest Cr concentration resulted in a 25% and 58% biomass reduction in *C. hirsuta* and *S. media* shoots, and an 81% biomass decrease in *P. annua* roots (Fig. 4b,h,d). Strong root growth inhibition has been previously observed in *Allium cepa* L. bulbs treated with Cr at concentrations between 50 and 200 μM Cr^27^. On the other hand, Cr showed a great potential in stimulating hormesis at intermediate concentrations (5–50 μM). *C. hirsuta* root and shoot dry weights/plant were on average three to fivefold higher in Cr-treated plants at 5–50 μM compared to the control. Biomass growth was more affected in shoots than in roots, as Cr was actively transferred to the aerial parts, resulting in concentrations 130% higher in shoots compared to roots (Table 1), again in agreement with reports directly relating this effect to metal accumulation levels^26^ and, to this extent, to our Cd data. *P. annua* and *S. media* also showed a clear increase in shoot biomass (128% and 115%, respectively) when treated with 5–25 μM Cr. In accordance with our results, other studies have reported the occurrence of beneficial effects of low doses of Cr. A study on *Mentha piperita* reported an increase of 51% and 71% in vegetative biomass, compared to control treatment, when applying 30 mg/kg and 60 mg/kg of Cr, respectively, to the soil^43^. *Solanum nigrum* shoot fresh weight increased by 38% compared to the control when treated with 1 μM Cr^44^. This research also showed that *S. nigrum* growth stimulation was favoured by enhanced absorption of essential nutrients as well as by increased antioxidant activity. In fact, it has been previously demonstrated^34,45^ that also in *P. annua* and *S. media*, grown in hydroponics with a wide range of metals (among which Cd, Cr, and Pb), the increase in antioxidant activity and polyphenols content was strictly linked to the metal concentration in the nutrient solution.

Cr enhanced the growth rate of all the studied species, with more leaves and more branches produced in the same cultivation period. *S. media* node number increased by 80% when plants were treated with 5–25 μM Cr. At similar doses, hormesis has been observed in other species, such as *Allium cepa*^27^, which showed maximum root stimulation at 6.25 μM Cr, or *Pisum sativum*^46^, which exhibited a 18% increase of root length when exposed to 20 μM Cr, compared to control plants.

In general, Cr treatments did not induce any effect on leaf area or photosynthetic pigments content, with exception of *S. media*, which showed a decrease in photosynthetic pigments content between 5 and 25 μM Cr (average 108.2 mg/kg FW), followed by an increase at 50 and 100 μM Cr, returning to levels comparable to the control treatment (average 128.9 mg/kg FW) (Supplementary Table 2). An increase in chlorophyll content was also reported for corn plants^47^ grown at 50–1000 μM Cr, showing, in agreement with present results, that at 100 μM Cr, the pigment content was equal to or slightly higher than that of the control. Photosynthetic pigment increase was also reported for several other plant species (such as *Picris divaricata*^6^ and *Dianthus carthusianorum*^48^) subjected to 1 to 3 μM Cd treatments. Pb treatments did not lead to a clear hormetic response in any of the chosen species (Fig. 5). Although this metal caused significative growth stimulation in *P. annua,* the tested concentrations (0.5–15 μM) were not sufficient to induce a complete hormetic response, as only a half inverted U-shaped curve could be observed (Fig. 5d–i). No information could be collected regarding the concentrations at which Pb is able to induce the maximum hormetic response or cause toxicity. In *C. hirsuta*, a slight increase in root and shoot biomass (not statistically significant, *p* = 0.162, *p* = 0.119) was detected between 1 and 5 μM Pb (Fig. 5a,b). The observed phenomenon could anyhow be ascribable to a hormetic response, as previously documented in maize^49^, in which a significant stimulation of shoot elongation (+ 27% compared to control) was detected in plants exposed to 5 µM Pb. Conversely, in *P. annua* and *S. media* a consistent increase in biomass was detected at the highest Pb concentration (15 μM); therefore, it can be speculated that a complete hormetic curve is possible but not under the tested Pb doses. Present results, in fact, are in agreement with data on *Arabis paniculata*^50^, which showed the highest plant biomass increase (+ 17% for shoots and + 43.2% for roots, compared to the control) at 48 µM Pb (3-times higher than the maximum concentration here tested), followed by a decrease at 97–386 µM Pb, thus resulting in a complete inverted U-shaped hormetic curve.

The presented results highlighted that, despite the intrinsic toxicity of the studied metals, these elements can also be beneficial to plants, if present at low concentrations in the nutrient solution. Considering the levels of Cd, Cr and Pb found in urban environments^30–32^, we can conclude that these concentrations are not harmful to plants, but instead can stimulate their growth inducing hormesis, although the specific thresholds at which each metal can cause hormesis or toxicity remain difficult to establish with certainty. Our results reported hormesis happening at a narrow concentration window (0.5–1 μM Cd, 5–50 μM Cr), hence, under environmental conditions, a change in element availability (i.e., due to a pH shift)^37^ can easily turn beneficial effects into toxicity. Root and shoot biomass can be considered the best traits indicating the insurgence of hormetic responses in plants, but the extent to which these parameters increase varies among species and types of stressors applied. In relation to trace metals, the present study showed that beneficial concentrations widely vary among tested metals and that interspecific diversities lead to different reactions in plants subjected to the same metal treatment. Furthermore, the effects of each metal were here singularly evaluated, whereas in urban environments several could be present simultaneously in the soil. Thus, interactions between different ions must also be taken into consideration. To further complicate the situation, urban plant communities are extremely diversified and rich in species. Nevertheless, in the light of the present findings, it can be speculated that urban metal pollution previously considered detrimental to plant organisms could instead be exactly the plus factor allowing urban plants to thrive.

## Supplementary Information

Supplementary Information.

## Data Availability

The datasets generated or analysed during this study are included in this article and its supporting materials.

## References

[1] Calabrese EJ (2015). Hormesis: Principles and applications. Homeopathy.

[2] Calabrese EJ (2015). Hormesis within a mechanistic context. Homeopathy.

[3] Calabrese EJ, Blain RB (2009). Hormesis and plant biology. Environ. Pollut..

[4] Berry R, López-Martínez G (2020). A dose of experimental hormesis: When mild stress protects and improves animal performance. Comp. Biochem. Phys. A.

[5] Jalal A (2021). Hormesis in plants: Physiological and biochemical responses. Ecotoxicol. Environ. Saf..

[6] Ying RR (2010). Cadmium tolerance of carbon assimilation enzymes and chloroplast in Zn/Cd hyperaccumulator *Picris divaricata*. J. Plant Physiol..

[7] Jalmi SK (2018). Traversing the links between heavy metal stress and plant signaling. Front. Plant Sci..

[8] Shahid M (2020). Trace elements-induced phytohormesis: A critical review and mechanistic interpretation. Crit. Rev. Environ. Sci. Technol..

[9] Jia L (2015). Hormesis effects induced by cadmium on growth and photosynthetic performance in a hyperaccumulator, *Lonicera japonica* Thunb. J. Plant Growth Regul..

[10] Ji KH (2019). Research progress on the biological effects of low-dose radiation in China. Dose-Response.

[11] Moghaddam NSA (2019). Hormetic effects of curcumin: What is the evidence?. J. Cell Physiol..

[12] Mathieu A (2016). Discovery and function of a general core hormetic stress response in *E. coli* induced by sublethal concentrations of antibiotics. Cell Rep..

[13] Agathokleous E, Feng ZZ, Penuelas J (2020). Chlorophyll hormesis: Are chlorophylls major components of stress biology in higher plants?. Sci. Total Environ..

[14] Brain P, Cousens R (1989). An equation to describe dose responses where there is stimulation of growth at low doses. Weed Res..

[15] Agathokleous E, Calabrese EJ (2021). Formaldehyde: Another hormesis-inducing chemical. Environ. Res..

[16] Agathokleous E, Kitao M, Harayama H, Calabrese EJ (2019). Temperature-induced hormesis in plants. J. For. Res..

[17] Kendig EL, Le HH, Belcher SM (2010). Defining hormesis: Evaluation of a complex concentration response phenomenon. Int. J. Toxicol..

[18] Agathokleous E, Kitao M, Calabrese EJ (2019). Hormesis: A compelling platform for sophisticated plant science. Trends Plant Sci..

[19] Agathokleous E, Kitao M, Calabrese EJ (2019). Hormetic dose responses induced by lanthanum in plants. Environ. Pollut..

[20] Cedergreen N, Felby C, Porter JR, Streibig JC (2009). Chemical stress can increase crop yield. Field Crop Res..

[21] Belz RG (2018). Herbicide hormesis can act as a driver of resistance evolution in weeds—PSII-target site resistance in *Chenopodium album* L. as a case study. Pest Manag. Sci..

[22] Islam F (2017). 2,4-D attenuates salinity-induced toxicity by mediating anatomical changes, antioxidant capacity and cation transporters in the roots of rice cultivars. Sci. Rep..

[23] Silva FML, Duke SO, Dayan FE, Velini ED (2016). Low doses of glyphosate change the responses of soyabean to subsequent glyphosate treatments. Weed Res..

[24] Agathokleous E, Calabrese EJ (2019). Hormesis can enhance agricultural sustainability in a changing world. Glob. Food Sec..

[25] Seth CS, Chaturvedi PK, Misra V (2007). Toxic effect of arsenate and cadmium alone and in combination on giant duckweed (*Spirodela polyrrhiza* L.) in response to its accumulation. Environ. Toxicol..

[26] Hajiboland R, Rad SB, Barceló J, Poschenrieder C (2013). Mechanisms of aluminum-induced growth stimulation in tea (*Camellia sinensis*). J. Plant Nutr. Soil Sci..

[27] Patnaik AR, Achary VMM, Panda BB (2013). Chromium (VI)-induced hormesis and genotoxicity are mediated through oxidative stress in root cells of *Allium cepa* L. Plant Growth Regul..

[28] Poschenrieder C, Cabot C, Martos S, Gallego B, Barceló J (2013). Do toxic ions induce hormesis in plants?. Plant Sci..

[29] Carvalho MEA, Castro PRC, Azevedo RA (2020). Hormesis in plants under Cd exposure: From toxic to beneficial element?. J. Hazard Mater..

[30] Salinitro M (2019). Heavy metals bioindication potential of the common weeds *Senecio vulgaris* L., *Polygonum aviculare* L. and *Poa annua* L. Molecules.

[31] Park B-J (2021). Assessment of heavy metal(loid)s pollution in urban soil at street tree planting sites in Chuncheon. Korean J. Soil Sci. Fertil..

[32] Al-Sudani IM, Al Lami MH, Al Obaidy AHMJ, Al-Rubay SMJ (2021). Spatial distribution of some heavy metals in urban soil of Western Iraq. Ann. Romanian Soc. Cell Biol..

[33] Salinitro M, Alessandrini A, Zappi A, Melucci D, Tassoni A (2018). Floristic diversity in different urban ecological niches of a southern European city. Sci. Rep..

[34] Salinitro M, van der Ent A, Tognacchini A, Tassoni A (2020). Stress responses and nickel and zinc accumulation in different accessions of *Stellaria media* (L.) Vill. in response to solution pH variation in hydroponic culture. Plant Physiol. Biochem..

[35] Hoagland DR, Arnon DI (1950). The water-culture method for growing plants without soil. Circ. Calif. Agric. Exp. Stn..

[36] Tüzen M (2003). Determination of heavy metals in soil, mushroom and plant samples by atomic absorption spectrometry. Microchem. J..

[37] Zia A (2018). Controls on accumulation and soil solution partitioning of heavy metals across upland sites in United Kingdom (UK). J. Environ. Manag..

[38] Xiong ZT, Peng YH (2001). Response of pollen germination and tube growth to cadmium with special reference to low concentration exposure. Ecotoxicol. Environ. Saf..

[39] Durenne B, Druart P, Blondel A, Fauconnier ML (2018). How cadmium affects the fitness and the glucosinolate content of oilseed rape plantlets. Environ. Exp. Bot..

[40] Duke SO, Cedergreen N, Velini ED, Belz RG (2006). Hormesis: Is it an important factor in herbicide use and allelopathy?. Outlooks Pest. Manag..

[41] Faller P, Kienzler K, Krieger-Liszkay A (2005). Mechanism of Cd2+ toxicity: Cd^2+^ inhibits photoactivation of Photosystem II by competitive binding to the essential Ca^2+^ site. BBA-Bioenergetics.

[42] Paunov M, Koleva L, Vassilev A, Vangronsveld J, Goltsev V (2018). Effects of different metals on photosynthesis: Cadmium and zinc affect chlorophyll fluorescence in *Durum* wheat. Int. J. Mol. Sci..

[43] Prasad A, Singh AK, Chand S, Chanotiya CS, Patra DD (2010). Effect of chromium and lead on yield, chemical composition of essential oil, and accumulation of heavy metals of mint species. Common. Soil Sci. Plant Anal..

[44] UdDin I, Bano A, Masood S (2015). Chromium toxicity tolerance of *Solanum nigrum* L. and *Parthenium hysterophorus* L. plants with reference to ion pattern, antioxidation activity and root exudation. Ecotoxicol. Environ. Saf..

[45] Salinitro M (2020). Production of antioxidant molecules in *Polygonum aviculare* (L.) and *Senecio vulgaris* (L.) under metal stress: A possible tool in the evaluation of plant metal tolerance. Int. J. Mol. Sci..

[46] Pandey V, Dixit V, Shyam R (2009). Chromium (VI) induced changes in growth and root plasma membrane redox activities in pea plants. Protoplasma.

[47] Sharma DC, Sharma C, Tripathi RD (2003). Phytotoxic lesions of chromium in maize. Chemosphere.

[48] Muszyńska E, Hanus-Fajerska E, Ciarkowska K (2018). Studies on lead and cadmium toxicity in *Dianthus carthusianorum* calamine ecotype cultivated in vitro. Plant Biol..

[49] Malkowski E (2020). Hormesis in plants: The role of oxidative stress, auxins and photosynthesis in corn treated with Cd or Pb. Int. J. Mol. Sci..

[50] Tang YT (2009). Lead, zinc, cadmium hyperaccumulation and growth stimulation in *Arabis paniculata* Franch. Environ. Exp. Bot..

