# Supplementary material for: Induction of hormesis in plants by urban trace metal pollution
Source: Sci Rep. 2021 Oct 13;11:20329. doi: 10.1038/s41598-021-99657-3 (PMC8514553; doi:10.1038/s41598-021-99657-3)
Supplement: Supplementary file 1 — Supplementary Information. [file 41598_2021_99657_MOESM1_ESM.pdf]

Supplementary Table 1. **Complete dataset related to Cd treatments**. Car: *Cardamine hirsuta;* poa: *Poa annua;* ste: *Stellaria media.* Met_root: Cd concentration in roots (mg/kg DW); met_shoot: Cd concentration in shoots (mg/kg DW); root_dw: root dry weight (g) per plant; shoot_dw: shoot dry weight (g) per plant; nodes: number of nodes per plant; chl_a: chlorophyll *a* (mg/kg FW); chl_b: chlorophyll *b* (mg/kg FW); carot: carotenoids (mg/kg FW); photo_pigm: total photosynthetic pigments (chl_a + chl_b + carot) (mg/kg FW); leaf_area: leaf area (cm^2). Data are presented in 5 replicates (n=5).

sample	species	treatment	met_root	met_shoot	root_dw	shoot_dw	nodes	chl_a	chl_b	carot	photo_pigm	leaf_area
car_cd_0	car	Control	0	0	0.002	0.328	23	252.077	103.116	73.602	142.931	15.308
car_cd_0	car	Control	0	0	0.006	0.411	25	274.02	124.776	82.367	160.388	17.579
car_cd_0	car	Control	0	0	0.002	0.251	25	295.284	134.961	93.197	174.481	14.386
car_cd_0	car	Control	0	0	0.002	0.205	20	263.545	108.344	79.746	150.545	16.59
car_cd_0	car	Control	0	0	0.004	0.265	24	230.99	96.052	75.777	134.273	14.1
car_cd_.5	car	0.5 uM	0	0	0.027	0.347	33	324.073	141.128	99.869	188.357	6.405
car_cd_.5	car	0.5 uM	0	0	0.014	0.548	39	299.954	129.544	93.436	174.311	14.112
car_cd_.5	car	0.5 uM	0	0	0.009	0.299	48	290.837	126.777	91.364	169.659	9.748
car_cd_.5	car	0.5 uM	0	0	0.017	0.429	44	299.341	129.916	92.351	173.869	8.749
car_cd_.5	car	0.5 uM	0	0	0.023	0.52	37	282.501	122.216	84.733	163.15	9.753
car_cd_.75	car	0.75 uM	0.019	1.733	0.041	0.794	44	288.995	103.045	93.451	161.83	12.862
car_cd_.75	car	0.75 uM	0.027	1.085	0.056	0.773	40	304.467	120.291	93.995	172.918	15.25
car_cd_.75	car	0.75 uM	0.03	1.236	0.071	0.65	34	292.622	111.505	91.656	165.261	16.209
car_cd_.75	car	0.75 uM	0.025	1.376	0.072	0.631	49	294.368	116.621	90.012	167	15.165
car_cd_.75	car	0.75 uM	0.025	1.127	0.066	0.684	41	282.658	106.063	89.165	159.295	15.355
car_cd_1	car	1 uM	0.015	3.827	0.016	0.793	31	307.921	115.409	100.298	174.543	16.01
car_cd_1	car	1 uM	0.031	2.92	0.006	0.964	32	255.208	89.462	86.695	143.788	18.007
car_cd_1	car	1 uM	0.033	3.456	0.004	1.044	28	291.641	115.408	99.476	168.842	18.222
car_cd_1	car	1 uM	0.028	3.715	0.005	1.032	31	253.037	92.29	85.344	143.557	13.082
car_cd_1	car	1 uM	0.03	3.289	0.016	1.02	33	259.366	91.769	85.041	145.392	11.302
car_cd_1.5	car	1.5 uM	0.04	4.84	0.032	0.884	34	296.21	112.766	93.221	167.399	15.804
car_cd_1.5	car	1.5 uM	0.045	5.251	0.002	0.515	23	250.888	95.089	82.46	142.812	9.684
car_cd_1.5	car	1.5 uM	0.049	4.91	0.007	0.785	32	277.785	112.782	87.559	159.375	15.182
car_cd_1.5	car	1.5 uM	0.045	5.71	0.021	0.663	31	271.267	107.826	80.709	153.267	8.068
car_cd_1.5	car	1.5 uM	0.044	5.201	0.003	0.6	29	283.565	111.504	89.649	161.573	14.728
car_cd_2	car	2 uM	0.049	5.868	0.006	0.367	21	287.505	108.472	91.168	162.382	13.93

car_cd_2	car	2 uM	0.044	5.789	0.003	0.318	15	258.35	98.851	81.833	146.344	7.747
car_cd_2	car	2 uM	0.05	5.251	0	0.214	18	267.125	102.065	84.662	151.284	13.47
car_cd_2	car	2 uM	0.037	5.427	0.002	0.415	25	270.911	110.489	85.213	155.538	16.292
car_cd_2	car	2 uM	0.038	4.929	0.004	0.376	22	270.973	104.969	85.719	153.887	14.418
poa_cd_0	poa	Control	0	0	0.022	0.348	7	307.341	123.438	92.189	174.323	2.366
poa_cd_0	poa	Control	0	0	0.012	0.311	12	325.262	143.648	96.882	188.597	5.244
poa_cd_0	poa	Control	0	0	0.02	0.243	11	332.138	143.207	95.787	190.377	4.205
poa_cd_0	poa	Control	0	0	0.014	0.435	15	318.428	108.218	99.113	175.253	5.556
poa_cd_0	poa	Control	0	0	0.041	0.404	14	332.838	131.1	103.248	189.062	6.018
poa_cd_.5	poa	0.5 uM	1.916	1.015	0.055	0.843	29	280.209	97.473	88.107	155.263	4.504
poa_cd_.5	poa	0.5 uM	2.627	1.244	0.071	0.473	20	283.958	98.023	89.691	157.224	4.978
poa_cd_.5	poa	0.5 uM	1.31	0.899	0.06	0.697	24	257.404	83.657	81.919	140.993	4.797
poa_cd_.5	poa	0.5 uM	2.146	0.724	0.059	1.065	27	297.809	107.429	90.924	165.387	7.257
poa_cd_.5	poa	0.5 uM	1.898	1.057	0.083	0.65	19	265.098	87.223	85.294	145.872	5.353
poa_cd_.75	poa	0.75 uM	3.341	1.198	0.087	0.893	26	273.159	94.524	83.821	150.501	7.27
poa_cd_.75	poa	0.75 uM	3.221	1.422	0.117	0.914	27	271.921	93.257	85.076	150.085	5.15
poa_cd_.75	poa	0.75 uM	3.066	1.556	0.086	0.969	30	265.171	88.612	81.338	145.04	6.444
poa_cd_.75	poa	0.75 uM	3.212	1.709	0.136	1.108	25	241.95	71.45	77.662	130.354	6.146
poa_cd_.75	poa	0.75 uM	2.956	1.43	0.133	0.764	23	261.586	87.672	84.52	144.593	4.524
poa_cd_1	poa	1 uM	6.422	5.024	0.045	0.685	24	281.423	100.099	87.51	156.344	6.49
poa_cd_1	poa	1 uM	5.532	4.513	0.06	0.832	25	258.226	93.338	81.954	144.506	8.32
poa_cd_1	poa	1 uM	5.667	3.861	0.032	0.995	27	254.975	87.748	79.598	140.774	6.787
poa_cd_1	poa	1 uM	6.121	5.033	0.065	1.214	29	251.807	88.293	82.423	140.841	5.422
poa_cd_1	poa	1 uM	5.744	4.103	0.105	0.713	24	300.539	111.2	95.819	169.186	6.499
poa_cd_1.5	poa	1.5 uM	10.119	8.072	0.061	0.548	16	269.387	92.652	77.594	146.544	5.321
poa_cd_1.5	poa	1.5 uM	9.698	7.417	0.045	0.81	21	222.07	69.598	70.624	120.764	4.985
poa_cd_1.5	poa	1.5 uM	10.037	7.203	0.054	0.636	19	280.614	93.797	81.32	151.91	4.412
poa_cd_1.5	poa	1.5 uM	9.976	6.796	0.042	0.881	21	270.055	89.33	83.483	147.623	5.982
poa_cd_1.5	poa	1.5 uM	9.758	8.12	0.038	0.525	19	286.277	101.71	84.781	157.589	6.34
poa_cd_2	poa	2 uM	21.434	13.902	0.014	0.278	9	304.741	112.615	91.254	169.537	4.567
poa_cd_2	poa	2 uM	18.061	12.178	0.012	0.382	11	314.056	121.393	95.812	177.087	4.588
poa_cd_2	poa	2 uM	19.03	13.967	0.024	0.405	12	304.049	112.711	89.507	168.756	4.587
poa_cd_2	poa	2 uM	20.13	13.895	0.021	0.301	10	283.113	91.728	85.975	153.605	4.303

poa_cd_2	poa	2 uM	17.185	14.806	0.02	0.387	13	284.975	101.576	83.766	156.772	5.406
ste_cd_0	ste	Control	0	0	0.125	1.729	7	211.975	73.685	68.492	118.05	3.833
ste_cd_0	ste	Control	0	0	0.157	1.712	10	196.002	67.777	67.777	110.519	5.934
ste_cd_0	ste	Control	0	0	0.108	1.427	10	215.993	74.51	74.51	121.671	7.139
ste_cd_0	ste	Control	0	0	0.123	1.467	10	214.323	74.712	64.809	117.948	5.467
ste_cd_0	ste	Control	0	0	0.133	0.804	9	221.582	77.739	66.871	122.064	4.649
ste_cd_.5	ste	0.5 uM	0.054	0	0.135	1.96	10	196.994	66.762	62.174	108.643	4.732
ste_cd_.5	ste	0.5 uM	0.062	0	0.155	1.707	10	218.641	73.236	67.851	119.909	7.162
ste_cd_.5	ste	0.5 uM	0.082	0	0.142	2.005	10	205.503	69.306	64.537	113.115	4.614
ste_cd_.5	ste	0.5 uM	0.07	0	0.152	0.958	8	209.869	73.336	64.969	116.058	2.454
ste_cd_.5	ste	0.5 uM	0.07	0	0.145	1.906	9	190.851	68.39	60.234	106.492	5.438
ste_cd_.75	ste	0.75 uM	0.085	1.674	0.101	0.517	9	222.643	75.809	69.963	122.805	3.303
ste_cd_.75	ste	0.75 uM	0.059	1.787	0.07	1.547	11	215.09	72.64	67.448	118.393	3.794
ste_cd_.75	ste	0.75 uM	0.062	1.718	0.105	1.219	10	217.043	72.138	69.709	119.63	3.467
ste_cd_.75	ste	0.75 uM	0.073	1.75	0.068	2.315	10	217.346	74.203	72.08	121.21	4.05
ste_cd_.75	ste	0.75 uM	0.062	1.744	0.073	1.675	11	194.698	64.801	63.252	107.584	4.107
ste_cd_1	ste	1 uM	44.147	2.259	0.171	1.599	11	208.329	69.125	64.793	114.082	7.727
ste_cd_1	ste	1 uM	31.078	3.007	0.18	2.55	12	229.849	78.939	72.31	127.033	5.21
ste_cd_1	ste	1 uM	29.083	2.934	0.166	0.782	9	219.974	77.356	65.802	121.044	4.096
ste_cd_1	ste	1 uM	36.766	2.767	0.123	1.019	10	191.819	63.536	59.837	105.064	4.821
ste_cd_1	ste	1 uM	29.41	2.888	0.194	2.045	10	211.79	71.094	64.118	115.667	5.236
ste_cd_1.5	ste	1.5 uM	51.149	2.996	0.122	0.932	9	263.183	104.646	80.11	149.313	1.911
ste_cd_1.5	ste	1.5 uM	54.31	2.208	0.101	1.485	11	247.637	91.981	76.45	138.689	3.986
ste_cd_1.5	ste	1.5 uM	49.384	3.626	0.096	0.788	9	280.441	115.767	85.656	160.621	3.59
ste_cd_1.5	ste	1.5 uM	52.508	3.082	0.114	1.289	11	212.796	68.839	65.863	115.833	5.743
ste_cd_1.5	ste	1.5 uM	47.642	2.914	0.115	1.951	11	160.881	50.111	50.399	87.13	4.424
ste_cd_2	ste	2 uM	61.628	4.55	0.015	0.658	9	276.649	102.264	82.635	153.849	5.083
ste_cd_2	ste	2 uM	69.744	4.665	0.077	0.71	9	237.815	83.742	72.636	131.398	4.724
ste_cd_2	ste	2 uM	71.505	4.252	0.039	0.808	11	253.706	92.041	74.968	140.238	5.675
ste_cd_2	ste	2 uM	71.009	4.879	0.043	1.275	10	257.563	92.47	77.214	142.416	6.494
ste_cd_2	ste	2 uM	65.561	4.226	0.083	0.869	11	231.316	80.787	70.313	127.472	5.494

Supplementary Table 2. **Complete dataset related to Cr treatments.** Car: *Cardamine hirsuta;* poa: *Poa annua;* ste: *Stellaria media.* Met_root: Cd concentration in roots (mg/kg DW); met_shoot: Cd concentration in shoots (mg/kg DW); root_dw: root dry weight (g) per plant; shoot_dw: shoot dry weight (g) per plant; nodes: number of nodes per plant; chl_a: chlorophyll *a* (mg/kg FW); chl_b: chlorophyll *b* (mg/kg FW); carot: carotenoids (mg/kg FW); photo_pigm: total photosynthetic pigments (chl_a + chl_b + carot) (μg/kg FW); leaf_area: leaf area (cm^2). Data are presented in 5 replicates (n=5).

sample	species	treatment	met_root	met_shoot	root_dw	shoot_dw	nodes	chl_a	chl_b	carot	photo_pigm	leaf_area
car_cr_0	car	Control	0.07	3.67	0	0.019	11	212.561	86.138	68.59	122.429	0.331
car_cr_0	car	Control	0.077	3.08	0	0.018	10	265.835	110.083	76.598	150.839	0.426
car_cr_0	car	Control	0.075	4.45	0	0.012	7	215.87	81.343	59.789	119.001	0.437
car_cr_0	car	Control	0.074	3.73	0.002	0.091	12	246.946	97.696	76.071	140.237	0.843
car_cr_0	car	Control	0.078	3.97	0.003	0.083	14	247.065	101.323	69.126	139.171	0.749
car_cr_5	car	5 uM	0.63	3.69	0.009	0.114	14	187.892	72.898	62.055	107.615	0.509
car_cr_5	car	5 uM	0.687	3.42	0.011	0.089	12	192.316	74.142	63.461	109.973	0.652
car_cr_5	car	5 uM	0.872	4.28	0.011	0.101	15	269.743	101.349	74.592	148.562	0.798
car_cr_5	car	5 uM	0.73	3.8	0.009	0.129	20	244.071	98.482	72.436	138.33	1.028
car_cr_5	car	5 uM	0.767	3.86	0.015	0.112	22	274.701	112.592	79.426	155.573	0.847
car_cr_10	car	10 uM	1.863	1.99	0.024	0.211	28	281.921	117.004	78.586	159.171	0.59
car_cr_10	car	10 uM	1.898	6.85	0.03	0.229	26	289.333	114.341	90.694	164.789	0.548
car_cr_10	car	10 uM	1.515	6.25	0.011	0.133	19	284.018	115.434	83.151	160.868	0.552
car_cr_10	car	10 uM	1.759	5.03	0.016	0.124	23	258.475	105.524	79.601	147.867	0.588
car_cr_10	car	10 uM	1.781	5.38	0.031	0.201	21	284.595	124.91	79.587	163.031	0.693
car_cr_25	car	25 uM	3.588	5.33	0.019	0.165	19	291.6	120.455	86.186	166.081	0.492
car_cr_25	car	25 uM	3.285	6.19	0.024	0.148	17	216.532	85.715	68.948	123.732	0.67
car_cr_25	car	25 uM	3.169	6.73	0.011	0.182	14	247.474	101.956	78.832	142.754	0.454
car_cr_25	car	25 uM	3.347	6.08	0.036	0.213	21	287.062	123.271	84.351	164.895	0.835
car_cr_25	car	25 uM	3.419	6.41	0.027	0.155	14	242.446	100.106	66.598	136.383	0.605
car_cr_50	car	50 uM	7.991	7.65	0.023	0.117	12	209.599	87.985	70.112	122.565	0.755
car_cr_50	car	50 uM	7.349	8.33	0.012	0.128	15	235.866	97.206	71.599	134.89	0.613
car_cr_50	car	50 uM	7.289	9.29	0.026	0.184	19	272.316	116.201	84.619	157.712	0.657
car_cr_50	car	50 uM	7.543	8.42	0.017	0.189	20	285.985	145.593	84.652	172.077	0.449
car_cr_50	car	50 uM	8.068	8.64	0.018	0.129	14	293.423	130.972	85.839	170.078	0.586
car_cr_100	car	100 uM	19.475	15.58	0.001	0.043	11	213.237	87.104	66.416	122.253	0.589
car_cr_100	car	100 uM	19.674	19	0.001	0.01	5	275.877	108.786	74.413	153.026	0.325

car_cr_100	car	100 uM	20.871	14.55	0	0.021	8	253.402	99.188	76.779	143.123	0.603
car_cr_100	car	100 uM	20.007	13.05	0	0.015	6	254.626	102.149	73.945	143.573	0.82
car_cr_100	car	100 uM	21.243	13.73	0.001	0.047	4	275.986	113.516	78.172	155.892	0.547
poa_cr_0	poa	Control	0.076	1.32	0.043	0.232	9	335.442	156.865	88.658	193.655	0.355
poa_cr_0	poa	Control	0.046	1.65	0.043	0.222	13	329.883	139.312	93.585	187.593	0.32
poa_cr_0	poa	Control	0.037	2.04	0.14	0.45	15	332.379	151.525	87.784	190.563	0.545
poa_cr_0	poa	Control	0.053	1.67	0.082	0.38	14	338.428	159.711	89.535	195.891	0.463
poa_cr_0	poa	Control	0.055	1.77	0.083	0.295	11	330.837	145.201	90.047	188.695	0.449
poa_cr_5	poa	5 uM	0.41	1.41	0.16	0.558	22	324.173	140.653	90.483	185.103	0.473
poa_cr_5	poa	5 uM	0.536	1.52	0.133	0.512	20	313.099	130.553	90.675	178.109	0.338
poa_cr_5	poa	5 uM	0.44	2.08	0.16	0.478	17	305.569	126.295	93.143	175.003	0.428
poa_cr_5	poa	5 uM	0.462	1.67	0.226	0.769	24	319.235	139.222	92.931	183.796	0.503
poa_cr_5	poa	5 uM	0.474	1.71	0.208	0.63	20	303.179	116.043	86.142	168.455	0.403
poa_cr_10	poa	10 uM	1.113	2.29	0.135	0.484	24	249.667	88.449	76.841	138.319	0.303
poa_cr_10	poa	10 uM	1.207	2.76	0.196	0.685	22	275.553	111.367	81.856	156.258	0.239
poa_cr_10	poa	10 uM	0.92	2.78	0.159	0.506	18	273.248	96.588	79.232	149.689	0.32
poa_cr_10	poa	10 uM	1.08	2.61	0.283	0.82	27	235.013	80.324	69.543	128.294	0.331
poa_cr_10	poa	10 uM	1.144	2.73	0.267	0.928	26	255.667	98.449	74.841	142.986	0.259
poa_cr_25	poa	25 uM	2.551	8.87	0.198	0.661	19	210.581	66.859	63.22	113.553	0.459
poa_cr_25	poa	25 uM	2.498	10.04	0.141	0.783	22	236.321	81.883	70.862	129.689	0.36
poa_cr_25	poa	25 uM	2.225	7.41	0.267	0.941	24	235.493	76.831	68.8	127.041	0.58
poa_cr_25	poa	25 uM	2.424	8.77	0.239	1.056	26	249.464	81.797	73.903	135.055	0.416
poa_cr_25	poa	25 uM	2.438	9.15	0.117	0.791	19	249.747	102.047	77.525	143.106	0.454
poa_cr_50	poa	50 uM	2.853	12.16	0.123	0.553	20	214.832	65.767	72.613	117.737	0.283
poa_cr_50	poa	50 uM	2.815	10.99	0.149	0.712	22	205.106	64.348	62.488	110.647	0.46
poa_cr_50	poa	50 uM	2.477	12.73	0.152	0.736	22	211.762	68.145	61.246	113.718	0.394
poa_cr_50	poa	50 uM	2.715	13.3	0.203	0.959	24	207.101	89.91	68.243	121.751	0.42
poa_cr_50	poa	50 uM	2.792	13.44	0.212	0.814	24	211.38	91.754	69.271	124.135	0.493
poa_cr_100	poa	100 uM	10.898	12.35	0.031	0.463	13	272.963	96.988	76.044	148.665	0.23
poa_cr_100	poa	100 uM	9.956	12.73	0.013	0.405	18	292.816	113.45	84.221	163.496	0.3
poa_cr_100	poa	100 uM	9.96	14.49	0.033	0.481	20	317.53	138.875	84.415	180.273	0.267
poa_cr_100	poa	100 uM	10.271	12.52	0.026	0.432	11	309.83	122.168	83.183	171.727	0.434
poa_cr_100	poa	100 uM	10.696	13.16	0.043	0.284	15	326.668	154.631	97.505	192.935	0.246

ste_cr_0	ste	Control	0.008	0	0.163	0.984	11	250.564	94.178	69.83	138.191	0.927
ste_cr_0	ste	Control	0.008	0	0.194	1.08	11	220.715	81.428	57.652	119.932	1.062
ste_cr_0	ste	Control	0.009	0	0.185	1.261	12	155.727	56.158	44.876	85.587	1.261
ste_cr_0	ste	Control	0.008	0	0.371	2.742	14	206.309	73.136	54.493	111.313	1.105
ste_cr_0	ste	Control	0.009	0	0.09	0.674	13	267.646	105.246	73.369	148.754	0.822
ste_cr_5	ste	5 uM	0.575	0	0.354	1.608	14	197.263	69.847	56.503	107.871	1.12
ste_cr_5	ste	5 uM	0.518	0	0.484	2.075	14	200.646	72.234	52.293	108.391	0.865
ste_cr_5	ste	5 uM	0.506	0	0.575	3.255	13	174.173	60.979	46.766	93.972	1.499
ste_cr_5	ste	5 uM	0.533	0	0.446	2.983	13	248.919	93.787	66.801	136.502	1.374
ste_cr_5	ste	5 uM	0.569	0	0.624	3.7	15	197.196	69.116	52.05	106.12	1.297
ste_cr_10	ste	10 uM	1.166	4.05	0.837	3.692	14	209.473	75.042	57.387	113.967	1.175
ste_cr_10	ste	10 uM	1.324	1.99	0.421	3.008	13	144.224	53.421	38.479	78.708	0.61
ste_cr_10	ste	10 uM	1.105	2.78	0.598	2.266	13	230.037	83.152	65.375	126.188	1.107
ste_cr_10	ste	10 uM	1.198	2.94	0.457	3.924	13	179.554	63.48	52.307	98.447	0.553
ste_cr_10	ste	10 uM	1.277	2.97	0.899	4.071	15	186.192	61.688	57.37	101.75	1.202
ste_cr_25	ste	25 uM	2.862	10.27	0.472	1.653	13	187.661	65.387	52.079	101.709	0.907
ste_cr_25	ste	25 uM	2.745	8.67	0.573	3.383	15	239.809	85.757	66.958	130.841	1.178
ste_cr_25	ste	25 uM	2.207	7.85	0.622	2.415	15	184.679	65.378	52.318	100.792	1.197
ste_cr_25	ste	25 uM	2.605	8.93	0.487	1.165	13	191.943	67.391	50.437	103.257	0.988
ste_cr_25	ste	25 uM	2.687	9.39	0.567	2.879	15	230.42	86.903	60.793	126.039	1.091
ste_cr_50	ste	50 uM	3.84	23.84	0.135	0.501	12	239.132	81.492	70.999	130.541	0.523
ste_cr_50	ste	50 uM	3.736	24.55	0.112	0.648	12	250.711	89.668	70.847	137.075	0.548
ste_cr_50	ste	50 uM	3.893	28.01	0.137	0.715	13	254.121	95.313	68.168	139.201	0.609
ste_cr_50	ste	50 uM	3.823	25.46	0.155	0.767	13	227.828	78.929	66.625	124.461	0.634
ste_cr_50	ste	50 uM	3.929	25.83	0.156	0.588	12	281.761	102.937	77.594	154.097	0.445
ste_cr_100	ste	100 uM	16.925	32.98	0.004	0.004	9	192.347	72.937	67.426	110.903	0.224
ste_cr_100	ste	100 uM	16.618	43.1	0.004	0.077	8	262.248	103.958	75.111	147.106	0.198
ste_cr_100	ste	100 uM	15.31	37.64	0.002	0.062	8	169.846	59.054	48.191	92.364	0.255
ste_cr_100	ste	100 uM	16.284	37.91	0.002	0.043	8	164.092	104.529	41.916	103.512	0.184
ste_cr_100	ste	100 uM	16.728	40.21	0.003	0.139	9	263.264	94.98	75.012	144.419	0.229

Supplementary Table 3. **Complete dataset related to Pb treatments.** Car: *Cardamine hirsuta;* poa: *Poa annua;* ste: *Stellaria media.* Met_root: Cd concentration in roots (mg/kg DW); met_shoot: Cd concentration in shoots (mg/kg DW); root_dw: root dry weight (g) per plant; shoot_dw: shoot dry weight (g) per plant; nodes: number of nodes per plant; chl_a: chlorophyll *a* (mg/kg FW); chl_b: chlorophyll *b* (mg/kg FW); carot: carotenoids (mg/kg FW); photo_pigm: total photosynthetic pigments (chl_a + chl_b + carot) (mg/kg FW); leaf_area: leaf area (cm^2). Data are presented in 5 replicates (n=5).

sample	species	treatment	met_root	met_shoot	root_dw	shoot_dw	nodes	chl_a	chl_b	carot	photo_pigm	leaf_area
car_pb_0	car	Control	0	0	0.002	0.02	7	416.492	150.121	112.011	226.208	2.142
car_pb_0	car	Control	0	0	0.01	0.083	14	340.891	141.545	90.095	190.844	6.162
car_pb_0	car	Control	0	0	0.006	0.024	6	410.373	144.956	113.342	222.89	3.29
car_pb_0	car	Control	0	0	0.016	0.162	11	309.02	137.737	84.335	177.031	2.456
car_pb_0	car	Control	0	0	0.01	0.053	24	333.17	130.212	90.22	184.534	2.027
car_pb_.5	car	0.5 uM	0	0	0.017	0.045	13	301.647	127.212	78.358	169.072	2.644
car_pb_.5	car	0.5 uM	0	0	0.012	0.125	15	299.357	111.616	81.217	164.063	6.645
car_pb_.5	car	0.5 uM	0	0	0.028	0.261	19	343.039	148.542	91.755	194.446	17.361
car_pb_.5	car	0.5 uM	0	0	0.009	0.036	10	269.01	105.547	68.019	147.525	2.798
car_pb_.5	car	0.5 uM	0	0	0.017	0.117	14	303.263	123.229	79.837	168.777	7.362
car_pb_1	car	1 uM	0.062	1.809	0.023	0.137	20	270.865	111.217	72.498	151.527	6.822
car_pb_1	car	1 uM	0.051	1.963	0.014	0.085	26	259.311	109.385	70.835	146.51	2.466
car_pb_1	car	1 uM	0.078	1.352	0.018	0.11	21	294.289	117.087	78.647	163.341	4.928
car_pb_1	car	1 uM	0.057	1.542	0.015	0.122	15	298.115	112.369	82.98	164.488	10.956
car_pb_1	car	1 uM	0.042	1.195	0.046	0.233	19	231.743	106.028	57.529	131.767	8.939
car_pb_5	car	5 uM	0.231	4.498	0.009	0.036	9	270.779	97.203	70.235	146.073	2.564
car_pb_5	car	5 uM	0.168	3.199	0.028	0.121	18	315.714	127.736	86.803	176.751	6.253
car_pb_5	car	5 uM	0.185	4.226	0.024	0.129	21	260.811	98.69	68.572	142.691	6.567
car_pb_5	car	5 uM	0.199	3.737	0.015	0.079	16	312.445	123.411	87.139	174.332	4.593
car_pb_5	car	5 uM	0.166	4.005	0.01	0.091	10	325.355	135.961	85.64	182.319	5.036
car_pb_7.5	car	7.5 uM	0.218	6.073	0.023	0.087	23	302.196	114.414	85.46	167.357	11.155
car_pb_7.5	car	7.5 uM	0.261	6.039	0.011	0.082	21	276.596	107.705	78.037	154.113	2.62
car_pb_7.5	car	7.5 uM	0.187	5.763	0.015	0.069	12	256.402	96.26	67.4	140.021	3.66
car_pb_7.5	car	7.5 uM	0.237	6.518	0.008	0.031	8	281.037	75.989	109.017	155.348	3.499
car_pb_7.5	car	7.5 uM	0.262	6.042	0.008	0.025	9	346.959	158.284	93.703	199.648	3.351
car_pb_15	car	15 uM	0.412	5.527	0.038	0.091	17	335.329	134.012	95.637	188.326	3.937

car_pb_15	car	15 uM	0.504	6.066	0.011	0.074	14	264.041	97.964	73.044	145.016	4.987
car_pb_15	car	15 uM	0.424	4.963	0.005	0.015	6	376.521	131.587	102.234	203.447	1.3
car_pb_15	car	15 uM	0.623	5.534	0.011	0.093	14	207.176	80.566	58.54	115.427	2.252
car_pb_15	car	15 uM	0.553	5.293	0.027	0.235	19	290.545	110.947	79.885	160.459	8.86
poa_pb_0	poa	Control	0	0	0.005	0.037	5	343.232	197.503	91.577	210.771	3.709
poa_pb_0	poa	Control	0	0	0.015	0.048	3	376.97	173.2	103.868	218.013	3.674
poa_pb_0	poa	Control	0	0	0.016	0.042	4	335.366	137.959	92.577	188.634	3.114
poa_pb_0	poa	Control	0	0	0.01	0.035	3	369.37	171.229	100.1	213.566	2.796
poa_pb_0	poa	Control	0	0	0.017	0.058	3	374.91	180.001	102.308	219.073	3.483
poa_pb_.5	poa	0.5 uM	0.435	0	0.022	0.088	5	64.657	369.246	71.207	168.37	4.53
poa_pb_.5	poa	0.5 uM	1.183	0	0.02	0.1	7	341.588	190.98	90.891	207.82	4.366
poa_pb_.5	poa	0.5 uM	1.163	0	0.029	0.112	6	393.793	215.683	108.856	239.444	3.605
poa_pb_.5	poa	0.5 uM	1.91	0	0.043	0.13	7	316.515	193.074	81.886	197.159	3.92
poa_pb_.5	poa	0.5 uM	0.276	0	0.033	0.113	7	348.983	152.749	96.678	199.47	3.906
poa_pb_1	poa	1 uM	3.052	0	0.024	0.106	8	370.442	188.227	97.791	218.82	3.593
poa_pb_1	poa	1 uM	2.303	0	0.025	0.107	6	334.119	147.885	93.328	191.777	5.023
poa_pb_1	poa	1 uM	2.269	0	0.065	0.218	10	333.319	130.506	100.717	188.181	4.632
poa_pb_1	poa	1 uM	3.041	0	0.079	0.156	10	341.744	238.418	65.737	215.3	3.537
poa_pb_1	poa	1 uM	1.815	0	0.056	0.238	10	180.736	180.736	96.815	152.762	4.408
poa_pb_5	poa	5 uM	4.709	0.21	0.031	0.132	6	341.766	178.049	81.957	200.591	5.418
poa_pb_5	poa	5 uM	4.072	0.19	0.017	0.071	5	396.391	181.609	111.805	229.935	2.73
poa_pb_5	poa	5 uM	5.255	0.205	0.022	0.187	8	369.233	168.69	104.697	214.207	5.324
poa_pb_5	poa	5 uM	4.458	0.187	0.044	0.171	8	357.652	139.246	98.78	198.559	4.59
poa_pb_5	poa	5 uM	4.107	0.238	0.035	0.121	8	330.646	176.184	90.767	199.199	3.79
poa_pb_7.5	poa	7.5 uM	9.544	0.413	0.041	0.182	7	166.354	157.994	52.489	125.612	4.903
poa_pb_7.5	poa	7.5 uM	6.214	0.465	0.069	0.24	8	338.801	149.138	100	195.98	5.78
poa_pb_7.5	poa	7.5 uM	8.239	0.583	0.053	0.191	10	367.549	151.878	101.767	207.065	5.077
poa_pb_7.5	poa	7.5 uM	6.274	0.647	0.08	0.263	10	221.157	69.96	137.032	142.716	4.341
poa_pb_7.5	poa	7.5 uM	7.463	0.595	0.069	0.249	10	265.636	92.671	77.662	145.323	4.547
poa_pb_15	poa	15 uM	17.042	1.08	0.052	0.193	8	25.16	8.321	7.909	13.797	6.099
poa_pb_15	poa	15 uM	15.899	1.385	0.067	0.228	9	282.4	109.176	82.073	157.883	5.816
poa_pb_15	poa	15 uM	14.302	1.411	0.049	0.239	10	233.339	79.043	69.069	127.15	5.502

poa_pb_15	poa	15 uM	18.344	1.077	0.078	0.254	10	232.406	78.27	65.807	125.494	4.305
poa_pb_15	poa	15 uM	12.6	1.41	0.06	0.243	9	232.305	76.577	69.847	126.243	4.067
ste_pb_0	ste	Control	0	0	0.047	0.347	10	202.878	79.144	55.166	112.396	3.438
ste_pb_0	ste	Control	0	0	0.033	0.247	10	269.245	101.122	78.736	149.701	3.8
ste_pb_0	ste	Control	0	0	0.15	0.698	10	227.469	82.069	66.316	125.284	7.211
ste_pb_0	ste	Control	0	0	0.13	0.704	10	160.157	55.273	43.215	86.215	5.455
ste_pb_0	ste	Control	0	0	0.194	0.922	10	207.961	77.887	56.425	114.091	5.605
ste_pb_.5	ste	0.5 uM	0	0.054	0.112	0.51	9	163.367	55.205	43.472	87.348	5.698
ste_pb_.5	ste	0.5 uM	0	0.062	0.318	0.686	10	210.168	76.878	56.882	114.643	5.096
ste_pb_.5	ste	0.5 uM	0	0.082	0.127	0.86	10	186.304	66.357	51.598	101.419	6.347
ste_pb_.5	ste	0.5 uM	0	0.07	0.218	1.1	10	195.613	71.694	55.083	107.463	5.531
ste_pb_.5	ste	0.5 uM	0	0.07	0.299	1.261	11	183.757	60.883	50.121	98.254	6.364
ste_pb_1	ste	1 uM	0.154	0.822	0.102	0.275	11	219.667	79.575	58.797	119.346	5.824
ste_pb_1	ste	1 uM	0.071	0.79	0.046	0.416	11	178.284	68.282	47.037	97.868	5.387
ste_pb_1	ste	1 uM	0.218	0.118	0.276	0.941	9	200.341	70.161	55.052	108.518	7.313
ste_pb_1	ste	1 uM	0.189	0.97	0.23	0.996	10	219.508	76.943	56.464	117.638	6.115
ste_pb_1	ste	1 uM	0.201	0.684	0.221	0.972	10	197.297	64.955	58.512	106.921	4.549
ste_pb_5	ste	5 uM	3.417	2.288	0.134	0.975	11	231.488	80.188	62.115	124.597	6.176
ste_pb_5	ste	5 uM	3.122	3.001	0.281	1.1	10	208.671	72.714	57.862	113.082	6.904
ste_pb_5	ste	5 uM	2.922	1.896	0.109	0.809	10	159.291	56.524	44.779	86.865	5.322
ste_pb_5	ste	5 uM	3.685	2.972	0.26	1.332	10	155.78	54.747	44.343	84.957	7.858
ste_pb_5	ste	5 uM	2.943	2.193	0.164	1.053	10	177.733	61.42	45.658	94.937	4.883
ste_pb_7.5	ste	7.5 uM	5.122	4.333	0.18	1.135	11	171.903	60.614	49.754	94.09	6.827
ste_pb_7.5	ste	7.5 uM	5.443	3.758	0.226	1.172	10	183.325	66.592	52.069	100.662	6.155
ste_pb_7.5	ste	7.5 uM	4.946	4.557	0.395	1.715	11	204.235	130.986	48.805	128.008	7.967
ste_pb_7.5	ste	7.5 uM	5.257	3.868	0.116	0.567	10	193.73	71.503	54.619	106.617	5.432
ste_pb_7.5	ste	7.5 uM	4.769	4.361	0.302	1.971	11	216.354	75.154	58.001	116.503	5.042
ste_pb_15	ste	15 uM	6.169	4.593	0.136	0.678	10	204.895	73.407	56.977	111.76	4.304
ste_pb_15	ste	15 uM	6.982	4.802	0.2	1.117	11	172.859	58.886	46.503	92.749	5.971
ste_pb_15	ste	15 uM	7.158	4.517	0.252	1.535	11	197.071	65.326	58.843	107.08	6.076
ste_pb_15	ste	15 uM	7.109	5.602	0.21	1.328	10	197.799	71.632	51.572	107.001	7.685
ste_pb_15	ste	15 uM	6.557	4.938	0.233	1.604	11	206.051	69.759	57.429	111.079	5.416

Supplementary Table 4. One-way ANOVA statistical analysis output related to data presented for Cd, Cr and Pb treatments. DF, degrees of freedom, SS, sum of squares, MS, mean of squares. Significance codes: (***), $p \leq 0.001$; (**), $p \leq 0.01$; (*), $p \leq 0.05$.

**Cadmium treatment (Table 1, Fig.2, Supplementary Table S1)**							
**Species**	**Parameter**		**DF**	**SS**	**MS**	**F value**	***P* value**
C. hirsuta	Metal root (mg/kg DW)	Sample	5	0.009859	0.001972	103.7	1.92e-15 ***
		Residuals	24	0.000456	0.000019		
C. hirsuta	Metal shoot (mg/kg DW)	Sample	5	153.44	30.688	394.3	<2e-16 ***
		Residuals	24	1.87	0.078		
C. hirsuta	Root DW (g/plant)	Sample	5	0.0120	0.0024	33.12	5.09e-10 ***
		Residuals	24	0.0018	0.0007		
C. hirsuta	Shoot DW (g/plant)	Sample	5	1.7233	0.3447	33.61	4.37e-10 ***
		Residuals	24	0.2461	0.0103		
C. hirsuta	Number of nodes/plant	Sample	5	1864.2	372.8	21.26	4.17e-08 ***
		Residuals	24	420.8	17.5		
C. hirsuta	Leaf area (cm^2)	Sample	5	123.1	24.617	3.371	0.019 *
		Residuals	24	175.3	7.303		
C. hirsuta	Photo. pigm. (mg/kg FW)	Sample	5	1724	344.9	2.919	0.0338 *
		Residuals	24	2835	118.1		
P. annua	Metal root (mg/kg DW)	Sample	5	1230.8	246.16	458	<2e-16 ***
		Residuals	24	12.9	0.54		
P. annua	Metal shoot (mg/kg DW)	Sample	5	681.2	136.24	511.5	<2e-16 ***
		Residuals	24	6.4	0.27		
P. annua	Root DW (g/plant)	Sample	5	0.029416	0.005883	20.79	5.17e-08 ***
		Residuals	24	0.006791	0.0003		
P. annua	Shoot DW (g/plant)	Sample	5	1.6251	0.3250	13.25	2.98e-06 ***
		Residuals	24	0.5888	0.0245		
P. annua	Number of nodes/plant	Sample	5	1173	234.59	30.27	1.28e-09 ***
		Residuals	24	186	7.75		
P. annua	Leaf area (cm^2)	Sample	5	14.80	2.960	2.801	0.0395*
		Residuals	24	25.36	1.057		
P. annua	Photo. pigm. (mg/kg FW)	Sample	5	5717	1143.4	10.23	2.42e-05***
		Residuals	24	2683	111.8		
S. media	Metal root (mg/kg DW)	Sample	5	22324	4465	408.4	<2e-16 ***
		Residuals	24	262	11		
S. media	Metal shoot (mg/kg DW)	Sample	5	79.59	15.919	224.6	<2e-16 ***
		Residuals	24	1.7	0.071		
S. media	Root DW (g/plant)	Sample	5	0.04445	0.008889	22.98	1.98e-08 ***
		Residuals	24	0.00928	0.000387		
S. media	Shoot DW (g/plant)	Sample	5	2.184	0.4368	1.678	0.178
		Residuals	24	6.248	0.2603		
S. media	Number of nodes/plant	Sample	5	5.9	1.180	1.057	0.408
		Residuals	24	26.8	1.117		
S. media	Leaf area (cm^2)	Sample	5	15.52	3.105	2.107	0.0995
		Residuals	24	35.36	1.473		
S. media	Photo. pigm. (mg/kg FW)	Sample	5	2524	504.9	2.713	0.0443 *
		Residuals	24	4466	186.1		
**Chromium Treatment (Table 1, Fig. 3, Supplementary Table S2)**							
**Species**	**Parameter**		**DF**	**SS**	**MS**	**F value**	***P* value**

C. hirsuta	Metal root	Sample	5	1464.2	292.83	2262	<2e-16 ***
	(mg/kg DW)	Residuals	24	3.1	0.13		
C. hirsuta	Metal shoot	Sample	5	469.7	93.94	56.66	1.67e-12 ***
	(mg/kg DW)	Residuals	24	39.8	1.66		
C. hirsuta	Root DW	Sample	5	0.0026831	0.000536	16	5.78e-07 ***
	(g/plant)	Residuals	24	0.0008049	0.000034		
C. hirsuta	Shoot DW	Sample	5	0.10683	0.021366	20.84	5.06e-08 ***
	(g/plant)	Residuals	24	0.02461	0.001025		
C. hirsuta	Number of	Sample	5	814.7	162.94	14.7	1.22e-06 ***
	nodes/plant	Residuals	24	266.0	11.08		
C. hirsuta	Leaf area	Sample	5	0.1408	0.02817	1.062	0.406
	(cm^2)	Residuals	24	0.6368	0.02653		
C. hirsuta	Photo. pigm.	Sample	5	2641	528.3	1.863	0.139
	(mg/kg FW)	Residuals	24	6807	283.6		
P. annua	Metal root	Sample	5	365.7	73.14	1866	<2e-16 ***
	(mg/kg DW)	Residuals	24	0.9	0.04		
P. annua	Metal shoot	Sample	5	728.5	145.7	312	<2e-16 ***
	(mg/kg DW)	Residuals	24	11.2	0.47		
P. annua	Root DW	Sample	5	0.12805	0.025609	11.92	7.17e-06 ***
	(g/plant)	Residuals	24	0.05157	0.002149		
P. annua	Shoot DW	Sample	5	1.038	0.20768	11.12	1.25e-05 ***
	(g/plant)	Residuals	24	0.448	0.01867		
P. annua	Number of	Sample	5	491	98.19	11.55	9.24e-06 ***
	nodes/plant	Residuals	24	204	8.50		
P. annua	Leaf area	Sample	5	0.1299	0.025982	4.659	0.0041 **
	(cm^2)	Residuals	24	0.1338	0.005576		
P. annua	Photo. pigm.	Sample	5	21499	4300	42.78	3.48e-11 ***
	(mg/kg FW)	Residuals	24	2412	100		
S. media	Metal root	Sample	5	953.1	190.62	2365	<2e-16 ***
	(mg/kg DW)	Residuals	24	1.9	0.08		
S. media	Metal shoot	Sample	5	6274	1255	424.3	<2e-16 ***
	(mg/kg DW)	Residuals	24	71	3		
S. media	Root DW	Sample	5	1.6551	0.3310	26.96	4.11e-09 ***
	(g/plant)	Residuals	24	0.2947	0.0123		
S. media	Shoot DW	Sample	5	40.86	8.172	17.65	2.38e-07 ***
	(g/plant)	Residuals	24	11.11	0.463		
S. media	Number of	Sample	5	113.4	22.673	27.21	3.75e-09 ***
	nodes/plant	Residuals	24	20.0	0.833		
S. media	Leaf area	Sample	5	8465	658.5	1.867	0.138
	(cm^2)	Residuals	24	3292	352.7		
S. media	Photo. pigm.	Sample	5	3.615	0.7229	20.33	6.39e-08 ***
	(mg/kg FW)	Residuals	24	0.853	0.0356		
**Lead treatment (Table 1, Fig. 4, Supplementary Table S3)**							
**Species**	**Parameter**		**DF**	**SS**	**MS**	**F value**	***P* value**
C. hirsuta	Metal root	Sample	5	0.9276	0.18551	114.2	6.33e-16 ***
	(mg/kg DW)	Residuals	24	0.039	0.00162		
C. hirsuta	Metal shoot	Sample	5	182.14	36.43	374.6	<2e-16 ***
	(mg/kg DW)	Residuals	24	2.33	0.10		
C. hirsuta	Root DW	Sample	5	0.0003540	7.07e-05	1.747	0.162
	(g/plant)	Residuals	24	0.0009726	4.05e-05		

C. hirsuta	Shoot DW (g/plant)	Sample	5	0.01224	0.002448	1.975	0.119
		Residuals	24	0.02974	0.001239		
C. hirsuta	Number of nodes/plant	Sample	5	26.2	5.233	0.18	0.968
		Residuals	24	699.2	29.133		
C. hirsuta	Leaf area (cm^2)	Sample	5	61.27	12.25	0.999	0.44
		Residuals	24	294.47	12.27		
C. hirsuta	Photo. pigm. (mg/kg FW)	Sample	5	6893	1379	2.714	0.0542
		Residuals	24	12193	508		
P. annua	Metal root (mg/kg DW)	Sample	5	834.8	166.96	124.9	2.28e-16 ***
		Residuals	24	32.1	1.34		
P. annua	Metal shoot (mg/kg DW)	Sample	5	6.373	1.2747	184.6	<2e-16 ***
		Residuals	24	0.166	0.0069		
P. annua	Root DW (g/plant)	Sample	5	0.010143	0.002028	10.18	2.51e-05 ***
		Residuals	24	0.004783	0.000199		
P. annua	Shoot DW (g/plant)	Sample	5	0.12816	0.025632	19.23	1.08e-07 ***
		Residuals	24	0.03199	0.001333		
P. annua	Number of nodes/plant	Sample	5	116.7	23.333	14.74	1.19e-06 ***
		Residuals	24	38.0	1.583		
P. annua	Leaf area (cm^2)	Sample	5	10.36	2.0719	3.958	0.00927 **
		Residuals	24	12.56	0.5234		
P. annua	Photo. pigm. (mg/kg FW)	Sample	5	37736	7547	7.468	0.000239 ***
		Residuals	24	24255	1011		
S. media	Metal root (mg/kg DW)	Sample	5	218.46	43.69	731.5	<2e-16 ***
		Residuals	24	1.43	0.06		
S. media	Metal shoot (mg/kg DW)	Sample	5	113.9	22.78	208.4	<2e-16 ***
		Residuals	24	2.62	0.109		
S. media	Root DW (g/plant)	Sample	5	0.05131	0.010261	1.446	0.244
		Residuals	24	0.17033	0.007097		
S. media	Shoot DW (g/plant)	Sample	5	2.114	0.4228	3.311	0.0205 *
		Residuals	24	3.065	0.1277		
S. media	Number of nodes/plant	Sample	5	1.867	0.3733	1.12	0.376
		Residuals	24	8.000	0.3333		
S. media	Leaf area (cm^2)	Sample	5	4.48	0.8952	0.676	0.645
		Residuals	24	31.76	1.3235		
S. media	Photo. pigm. (mg/kg FW)	Sample	5	942	188.5	0.907	0.493
		Residuals	24	4985	207.7		
